# Uniaxial Strain-Induced Stacking Order Change in Trilayer Graphene

**DOI:** 10.1021/acsami.3c19101

**Published:** 2024-01-31

**Authors:** Aditya Dey, Ahmad Azizimanesh, Stephen M. Wu, Hesam Askari

**Affiliations:** † Department of Mechanical Engineering, University of Rochester, New York 14627, United States; ‡ Department of Electrical and Computer Engineering, University of Rochester, Rochester, New York 14627-0001, United States

**Keywords:** Trilayer graphene, Strain engineering, Stacking order change, Atomistic simulations, Raman measurements

## Abstract

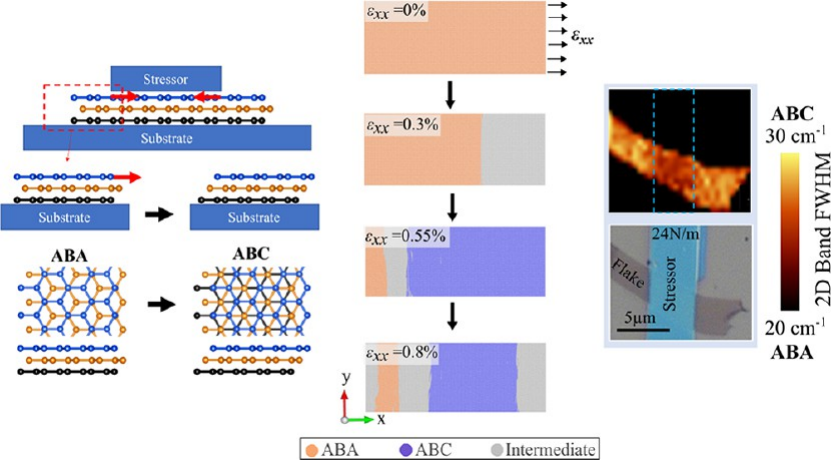

The layer stacking order in two-dimensional heterostructures, like graphene, affects their physical properties and potential applications. Trilayer graphene, specifically ABC-trilayer graphene, has captured significant interest due to its potential for correlated electronic states. However, achieving a stable ABC arrangement is challenging due to its lower thermodynamic stability compared to the more stable ABA stacking. Despite recent advancements in obtaining ABC graphene through external perturbations, such as strain, the stacking transition mechanism remains insufficiently explored. In this study, we unveil a universal mechanism to achieve ABC stacking, applicable for understanding ABA to ABC stacking changes induced by any mechanical perturbations. Our approach is based on a novel strain engineering technique that induces interlayer slippage and results in the formation of stable ABC domains. We investigate the underlying interfacial mechanisms of this stacking change through computational simulations and experiments. Our findings demonstrate a highly anisotropic and significant transformation of ABA stacking to large and stable ABC domains facilitated by interlayer slippage. Through atomistic simulations and local energy analysis, we systematically demonstrate the mechanism for this stacking transition, that is dependent on specific loading orientation. Understanding such a mechanism allows this material system to be engineered by design compatible with industrial techniques on a device-by-device level. We conduct Raman studies to validate and characterize the formed ABC stacking, highlighting its distinct features compared to the ABA region. Our results contribute to a clearer understanding of the stacking change mechanism and provide a robust and controllable method for achieving stable ABC domains, facilitating their use in developing advanced optoelectronic devices.

## 1 Introduction

Graphene, a two-dimensional (2D) carbon allotrope with remarkable electronic and mechanical properties, has attracted significant interest since its discovery. Driven by the quest for uncovering the distinct properties of graphene, many of its multilayered counterparts such as bilayer graphene (BLG) and trilayer graphene (TLG)^1−3^ have been studied. The multilayered stacking configurations demonstrate unique properties that differ from those observed in single-layer graphene. The emergence of these properties is linked to the interlayer interactions of atoms that affect the intralayer electronic behavior. For instance, BLG exhibits distinct characteristics such as tunable band gap, interlayer coupling, moiré patterns, and different optical properties^4,5^ that are absent in monolayer graphene. Similar properties are expected to emerge when transitioning from two layers to three in TLG, offering a rich landscape of properties. These properties arise from the diverse stacking types of trilayer graphene that influence its electronic behavior, optical response, and interlayer interactions.^6−8^ The crystallographic arrangement in TLG exhibits two main stacking types: the Bernal stacking order (ABA) and the rhombohedral stacking order (ABC).^9^ In the ABA-trilayer graphene (ABA-TLG) arrangement, the carbon atoms in the second layer are situated directly above the carbon atoms in the first layer, resulting in a hexagonal lattice. In contrast, the ABC-trilayer graphene (ABC-TLG) order places the carbon atoms in the third layer above the centers of the hexagons formed by the carbon atoms in the first two layers. These distinct stacking patterns in TLG give rise to different electronic and structural properties, impacting band structure, electronic transport, optical properties, interlayer interactions, and potential for correlated electronic states.^9,10^

Both ABA- and ABC-TLG have attracted considerable attention due to their unique properties and potential applications. ABA-TLG behaves as a semimetal and exhibits band overlapping that can be fine-tuned through an electric field.^11^ Interestingly, unlike its mono- and bilayer counterparts, it remains metallic even under a perpendicular electric field, making its transport characteristics a subject of ongoing research.^12,13^ On the other hand, ABC-TLG possesses semiconducting properties, and its band gap can be adjusted through electrical stimulation.^14^ ABC graphene demonstrates unique features compared to ABA due to its interesting optoelectronic properties.^14,15^ It features an electric field tunable band gap^14^ and showcases van Hove singularities near the band edge, leading to a divergent density of states.^16^ This configuration has also been shown to exhibit Mott insulator behavior, superconductivity, and ferromagnetism.^17−19^ These unique characteristics make ABC graphene a promising platform for advanced technologies in areas such as optoelectronics and quantum computing.

**Figure 1:**
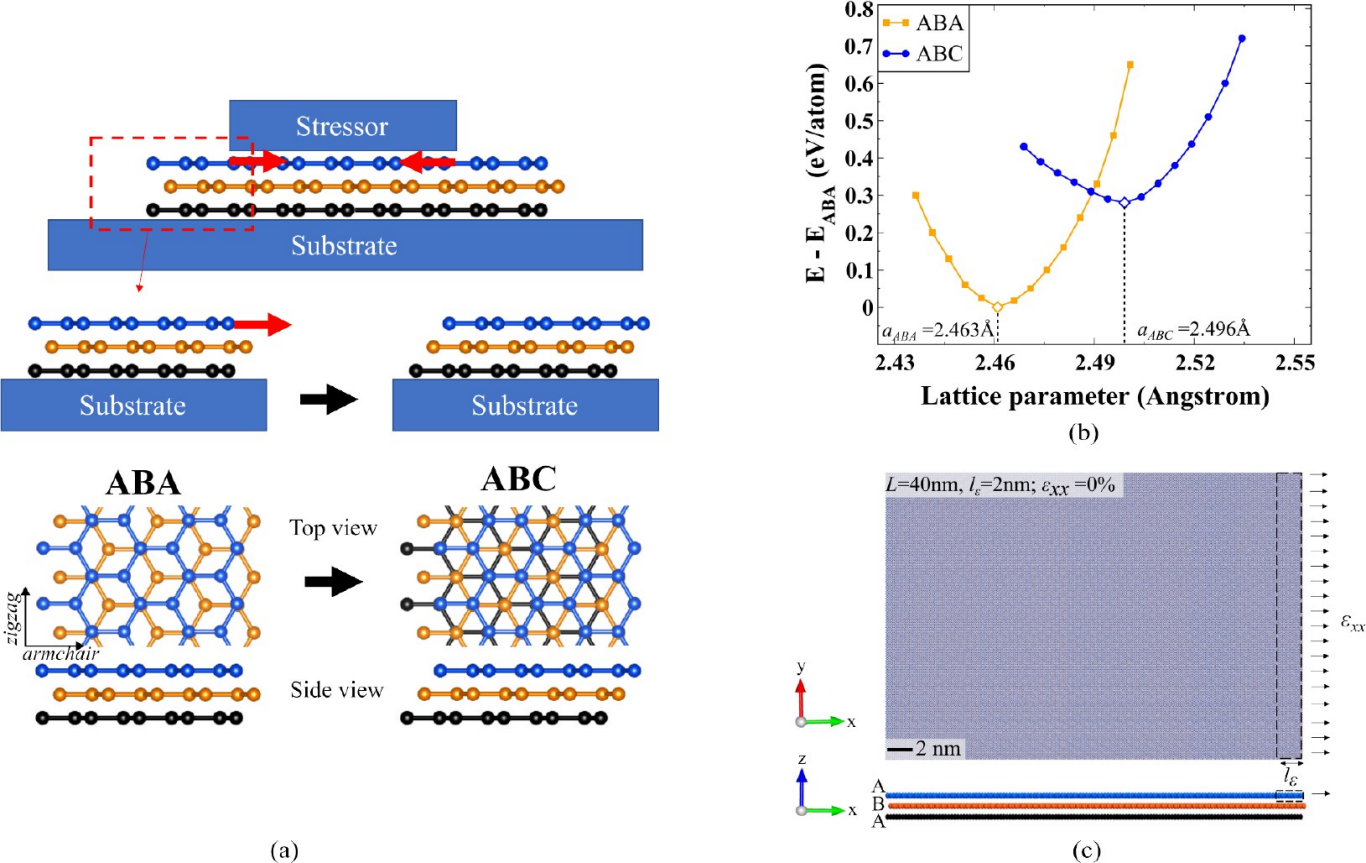
Device setup schematic. (a) Schematic representation of the strain-engineered device featuring a localized stressor applied to the top layer of ABA graphene. The stressor when deposited on the top layer of ABA-TLG induces compressive strain in the region directly under the stressor. Simultaneously, the edges of the top layer located outside the stressor experience uniaxial tension due to this phenomenon (depicted by red arrows). This tensile strain facilitates the transition from ABA to ABC stacking. (b) Comparative energy analysis of ABA and ABC graphene, highlighting the relative stability of Bernal stacked (ABA) compared to rhombohedral (ABC) trilayer graphene. (c) Atomistic model utilized for molecular statics (MS) simulations to employ the strain engineering technique on ABA graphene. The model incorporates uniaxial tensile strain applied at the right edge of the ABA flake along the armchair axis, as indicated by black arrows. The tab region, with a width of *l*_ε_ = 2 nm (5% of the flake length), is shown by the black dotted box.

Efforts to fabricate and explore the properties of ABC-TLG have involved various synthesis techniques, such as chemical vapor deposition and mechanical exfoliation yielding thin films of ABC graphene.^20,21^ However, achieving stable ABC stacking in trilayer graphene is challenging due to its lower thermodynamic stability compared to the more stable ABA stacking configuration.^11,22^Figure 1(b) presents a comparative total energy analysis of pristine ABA and ABC graphene using DFT simulations (see the “Methods” section), supporting the lower energy of Bernal-stacked TLG compared to the rhombohedral stacking. Postgrowth treatments, such as intercalation or surface functionalization are often necessary to transform the initially formed ABA structure into the desired ABC stacking.^22^ However, maintaining the stability of ABC-TLG presents additional complexities influenced by factors like temperature conditions, substrate selection, and synthesis methodologies that potentially trigger reversion to ABA stacking.^23,24^ Several techniques have been explored to stabilize or alter the stacking configuration in TLG, including strain,^25^ laser irradiation,^26^ electric fields,^11^ and doping.^27^ For instance, Nery et al. proposed a theoretical model demonstrating the transformation from ABA to ABC by applying shear deformation,^28^ that was experimentally implemented by Yang et al.^25^ Despite advancements, these methods have intrinsic limitations, with obtained ABC domains degrading upon the removal of applied constraints, such as encapsulation on the top layer. Other attempts involve manipulating ABA/ABC domain walls using the atomic force microscope (AFM) tip^29^ as well as using metal contact deposition to induce strain from evaporated films.^30^ However, these methods are quite uncontrollable and yield a repeated mixture of ABA and ABC domains across the flake rather than a high yield of pure ABC stacking.

While the mentioned techniques have been able to induce the transition through perturbations like mechanical strain and electric field, it is important to note that this transition remains a complex and intricate phenomenon. The missing piece is a clear understanding of the mechanism and the driving interfacial phenomena controlling this transformation. Previous works have explored methods to attain stacking change, but the reasons for certain mechanical deformations resulting in mixed and unstable ABC domains are unknown. Given the close energetic proximity of ABA and ABC stacking, the factors leading to mixed and unstable ABC domains following specific mechanical deformations may hinge on a precise understanding of the underlying mechanism. Thus, apart from exploring a more robust and controllable technique, there is a need for a systematic investigation into the crystal orientation mechanism and the driving forces that govern the transition to ABC-TLG. In this work, we present a stacking transition mechanism applicable to comprehend the transformation induced by any mechanical deformations. Our approach employs a stressor stripe to strain a localized area on the top layer of ABA graphene, leading to a stacking change to ABC-TLG beyond the stressor region. We use this strain engineering approach to achieve stable ABC domains and understand the underlying procedure causing the ABA to ABC transformation using atomic-scale simulations and experiments to validate or characterize the obtained configurations.

## 2 Methods

### 2.1 MS Simulations

We perform molecular statics (MS) simulations using the LAMMPS open-source software^31^ to model TLG structures. We set the initial atomic model thickness to 6.68 Å to account for the interlayer distance in the ABA-TLG system.^3,8^ We study three different lengths of TLG flake (40, 100, and 200 nm) while maintaining a constant width of 50 nm for all of them to examine the dependency of our results on the size of the structure. The models have free surface boundary conditions along the loading direction (armchair, *x*-axis in Figure 1), enabling us to consider the aperiodic crystal geometry resulting from strain applied to the topmost layer. We implement a periodic boundary along the zigzag direction (*y*-axis in Figure 1). The periodic boundary condition along the zigzag direction prevents edge effects and simulates an effectively infinite TLG sheet, ensuring an accurate examination of strain-induced changes without artificial boundary interference. We have a large vacuum space of 50 Å along the out-of-plane direction (*z*-axis in Figure 1).

We utilize a reactive empirical bond order (REBO) potential,^32^ while for the interlayer van der Waals interaction, we employ a registry-dependent Kolmogorov–Crespi (KC) potential^33^ for the intralayer covalent bonds, which has been used previously.^34^ We fully relax the model using a conjugate gradient (CG) minimization algorithm to obtain the configuration with the lowest energy^35^ before applying loads. Subsequently, we conduct MS simulations at *T* = 0 K to apply a constant incremental uniaxial strain along the defined region in the top layer. We perform energy minimization at each loading step to keep atoms as close as possible to their stationary configuration and avoid dynamic loading. We fix the atoms in the bottom layer to mimic the condition where the bottom layer is attached to the substrate underneath assuming perfect bonding with the substrate. Meanwhile, all atoms in the top two layers, except for the tab region on one side (right side in the model shown in Figure 1(c)), are allowed to freely relax, facilitating the propagation of in-plane and out-of-plane strain. To analyze the tab samples and visualize the strain distribution in the films, we utilize the Ovito open visualization tool.^36^ We extract the local strain information and its spatial distribution across the film thickness using the “Atomic strain” feature in Ovito.^36^

### 2.2 DFT Calculations

The real space hexagonal unit lattice cell of the ABA-TLG structure is modeled in the ATOMISTIX TOOLKIT (QuantumATK) commercial package.^37^ We then perform first-principles simulations to obtain a fully relaxed ABA trilayer graphene system. We use the framework of generalized gradient approximation (GGA)^38,39^ embodied in the Quantum Espresso open-source package^40^ for conducting this calculation. The GGA, along with the Perdew–Burke–Ernzerhof (PBE) form, serves as the exchange-correlation functional with ultrasoft pseudopotentials.^41,42^ We account for the van der Waals interaction using the semiempirical Grimme functional (DFT-D2 (Grimme-D2)),^43^ also employed in earlier works.^44−47^ The wave functions are expanded by using a plane wave basis set with a kinetic energy cutoff of 40 Ry (544 eV) and an energy cutoff of 450 Ry (6123 eV) for charge density (and the self-consistent field potential). It is important to note that the energy cutoff defines the maximum energy for the plane wave basis set, ensuring that the basis set contains sufficient wave functions to accurately describe the electronic structure of the system. On the other hand, the charge density cutoff limits the Fourier components of the charge density and impacts the accuracy of the electronic structure calculations. These parameters are crucial for achieving convergence in DFT calculations. We employ a 12 × 12 × 1 *k*-point grid within the Monkhorst–Pack^48^ scheme to sample the Brillouin zone. We optimize the structures until all the atomic forces are less than 0.01 eV/Å. The in-plane lattice constants are relaxed with an out-of-plane vacuum space of 25 Å to avoid interaction between the periodic images. We compute the phonon dispersion spectra of the obtained MS-simulated ABA and ABC structures by employing self-consistent density functional perturbation theory (DFPT).^49,50^ In this method, we first compute the dynamical matrices on a sufficient *q*-point grid. The interatomic constant used in calculating the phonon dispersion and phonon density of states is computed from the Fourier interpolation of the dynamical matrices.

### 2.3 Experimental Techniques

Graphene samples were obtained through mechanical exfoliation from the bulk crystal onto flat SiO_2_ substrates using the standard tape exfoliation method.^51^ To enhance the adhesion between the graphene flakes and the substrate prior to exfoliation, SiO_2_/Si substrates underwent plasma cleaning in an O_2_ environment.^52^ To eliminate poorly adhered flakes and any residual tape, the samples were subjected to a 45 min ultrasonic bath in isopropyl alcohol (IPA). In order to determine the stacking order and the strain distribution in TLG samples, Raman spectroscopic mapping was performed before and after the application of the stressor layer. Raman microscopy has proven to be a valuable technique for investigating strain and stacking order in graphene.^53^ The distinction in Raman signatures between ABA and ABC TLG has been extensively studied in the past^7,9^ and is utilized in this study to identify the stacking order. Raman spectroscopy was carried out at room temperature using a WiTec Alpha300R Confocal Raman microscope with a 532 nm laser.

We employed direct-write laser photolithography, utilizing an S1805 photoresist development process, to create a 5 μm wide stripe pattern on the graphene samples. This geometry of the evaporated stressors has been used previously to create uniaxial strain in 2D materials.^54^ The evaporated stressors deposited onto the graphene flakes consisted of Ti (5 nm)/MgF_2_ (*X* nm)/Al_2_O_3_ (10 nm) using an e-beam evaporator operating at a base pressure of 5 × 10^–6^ Torr. To prevent any physical damage to the crystal structure of the graphene samples and enhance the adhesion between the stressor and the flakes, the Ti layer was evaporated at a rate of 0.1 Å/s. The thickness of the MgF_2_ layer was varied between 10 and 180 nm to control the film force (film stress × film thickness) of the stressor and subsequently the strain in the graphene samples.^55^ The top Al_2_O_3_ layer served as protection for the stressor film against potential humidity-induced relaxation. Subsequently, the sample was soaked in a solvent (Remover PG) to remove the photoresist, resulting in the patterned stripe stressors. Finally, Raman mapping was conducted once again following the deposition of the stressor layer. To calculate the intrinsic stress of the evaporated films, the radius of curvature is measured on a precleaned glass coverslip before and after deposition using a surface profilometer.^56^ The variation in the radius of curvature before and after deposition indicates the stress present in the film, as determined by the Stoney equation.^57^

## 3 Results and Discussion

### 3.1 Atomistic Model and Mechanical Behavior at the Interface

The structure used to obtain ABC stacking is shown in Figure 1(a). The procedure involves depositing a stressor with compressive strain onto a localized area of the top layer of an ABA-stacked TLG flake. As the stressor relaxes, it transfers uniaxial tensile strain to the edge of the stressor-deposited region. This induced strain in the top layer ultimately causes interlayer sliding, leading to a change in the stacking order of the flake beyond the stressor. To begin, we obtain the relaxed crystal structure of the Bernal-stacked (ABA) TLG using density functional theory (DFT, see methods). The resulting in-plane lattice constant (*a*) and interlayer distance (*d*) of the relaxed ABA structure as obtained by DFT are 2.463 and 3.344 Å, respectively. We have included a comparison of the lattice parameters of ABA-TLG with experimental data and results from other DFT methods in Table S1. These comparisons reveal that our calculations, which incorporate van der Waals (vdW) corrections in DFT, provide a more accurate prediction of lattice constants in similar stacked materials. This underscores the significance of considering the vdW term when computationally studying such structures. These relaxed lattice parameters are then utilized to construct large ABA-TLG flakes of various dimensions in MS, including 40, 100, and 200 nm to obtain a computational trend toward the flake dimensions used in our experiments. Figure 1(c) displays a snapshot of the relaxed and unstrained ABA flake (40 nm) obtained through MS simulations. In our atomistic simulations, we model the flake region outside the stressor because this area is expected to show stacking transition due to the tensile strain induced by the stressor edge. We start from the initially relaxed ABA structure and introduce a tab region at one edge of the flake (right side in our model, Figure 1(c)). This designated rigid tab region is used to employ uniaxial tension in the ABA top layer along armchair axis. The representative tab region percentages of flake lengths are 2.5%, 5%, 7.5%, and 10%. We present the outcomes corresponding to the 5% case in the main text to streamline our discussion (see Supplementary Section II). The applied external deformation stretches the weak interlayer van der Waals (vdW) bonds and ultimately causes sliding of the two layers leading to a mechanically induced stacking order change in the system.

**Figure 2:**
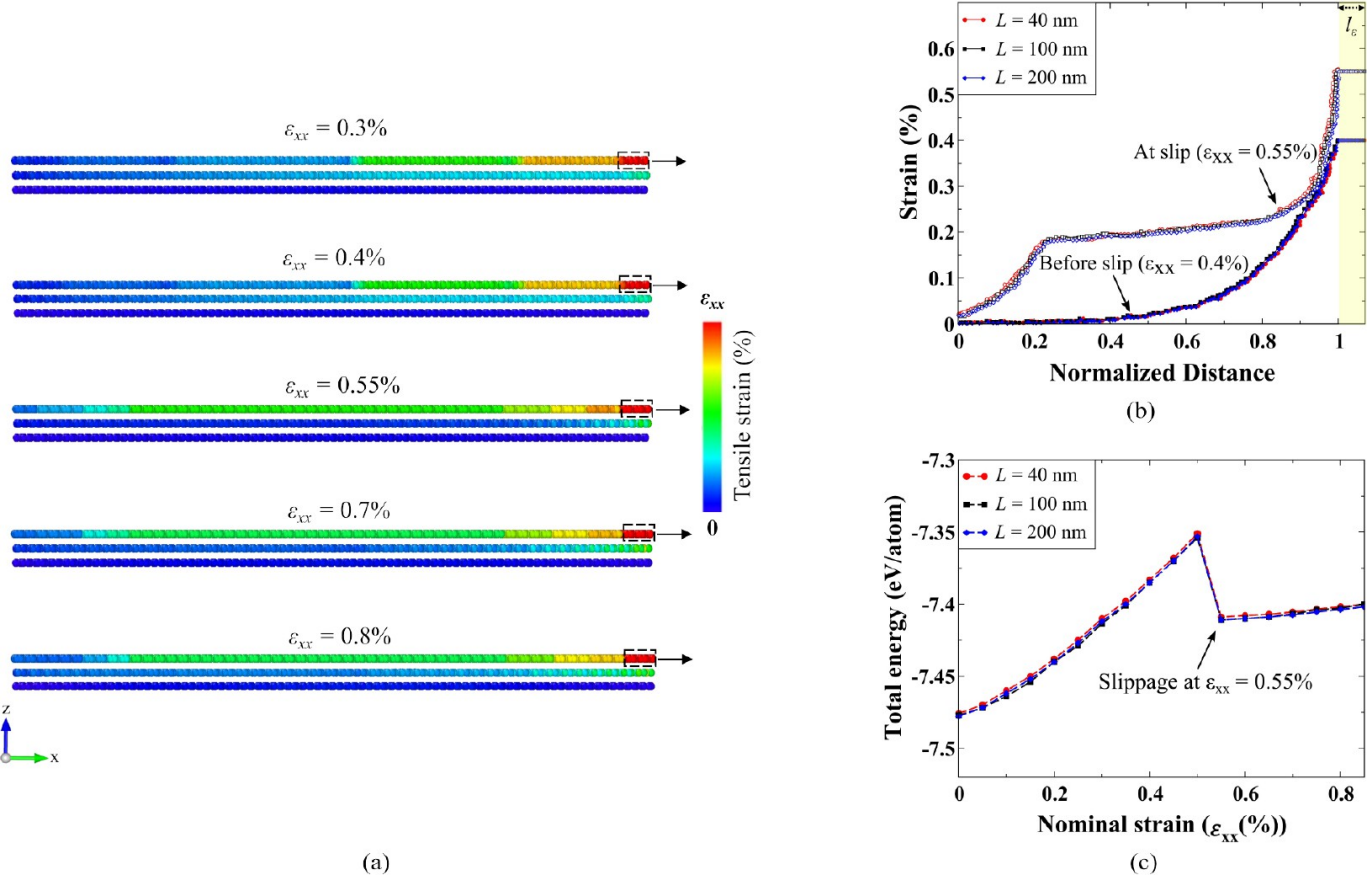
Strain distribution and strain profile. (a) Visualization of layer-by-layer atomic strain distribution from incremental tensile strain on the tab region, shown for the 40 nm flake. Tab region with *l*_ε_ = 2 nm (5% of the flake length) is shown by a black dotted box. (b) Strain profile of the top layer upon straining from the right edge for different lengths (normalized distances). Preslippage in-plane strain follows monotonous decay (shown for ε_*xx*_ = 0.4%). At slippage (ε_*xx*_ = 0.55%), strain magnitude plateaus beyond the tab region, and quickly decays traversing further to the opposite end. (c) Plot showing total energy variation with applied strain in the top layer for different flake widths. Significant energy drop at 0.55% strain indicates some structural reconfiguration due to the slippage.

We investigate the in-plane longitudinal strain profile of the top layer and analyze the layer-by-layer local strain. This reveals that for smaller ε_*xx*_ magnitudes, strain propagates in the top layer following a monotonous decay and becomes negligible toward the free end of the flake. This is easily visualized from the atomic strain contour plot for the two cases (ε_*xx*_ = 0.3% and 0.4%) shown in Figure 2(a) as well as from the top layer strain profile (ε_*xx*_ = 0.4%) shown in Figure 2(b). We also observe that the strain transfers to the second layer through interlayer vdW forces. Note that we report engineering strain in our results and we find that it closely matches atomic strain computed in the tab region (*l*_ε_). It indicates that the two measures of strain are consistent. Additionally, we detect no strain transfer to the bottom layer due to the fixed boundary conditions imposed on it.

We observe a transition in strain propagation occurring at and beyond ε_*xx*_ = 0.55% strain magnitude. The strain profile of the top layer is more intensified compared to the second layer as shown in Figure 2(b). This distinct behavior indicates that approximately around this critical strain magnitude, the interlayer shear strength mediated by the vdW bonds becomes insufficient to withstand further load leading to interlayer slippage. This measure of interlayer slippage is consistent with previously obtained values for interfaces of heterostrained stacked graphene systems using computations and experiments.^58−60^ As the layers undergo slipping, the limited strain transfer in the normal direction causes the applied deformation to propagate predominantly in the top layer. As a result, we observe a significant increase in the strain profile of the top layer while the second layer releases its accumulated strain as shown in Figure 2(a).

The sudden decrease in total energy per atom (TE/atom) of the system with respect to the applied strain as shown in Figure 2(c) indicates that the system relaxes to a local energy minimum at around ε_*xx*_ = 0.55%. This rearrangement is facilitated by a restructuring of the crystal lattice that may result in the formation of various new stacking configurations as seen in bilayer 2D materials.^59,61^ The nature of this transformation is unknown which we examine in the next sections. Applying further strain after the slippage event results in a slow but continuous rise in total energy and a gradual reinitiation of strain transfer to the second layer as shown in Figure 2(a) for ε_*xx*_ = 0.8%. It is also interesting to observe that the total energy and strain propagation profile remain consistent across various flake lengths (Figure 2) and width of the tab region (see Supplementary Section II).

### 3.2 Evolution of the Stacking Order Change

#### 3.2.1 Identification of Local Stacking Order

**Figure 3:**
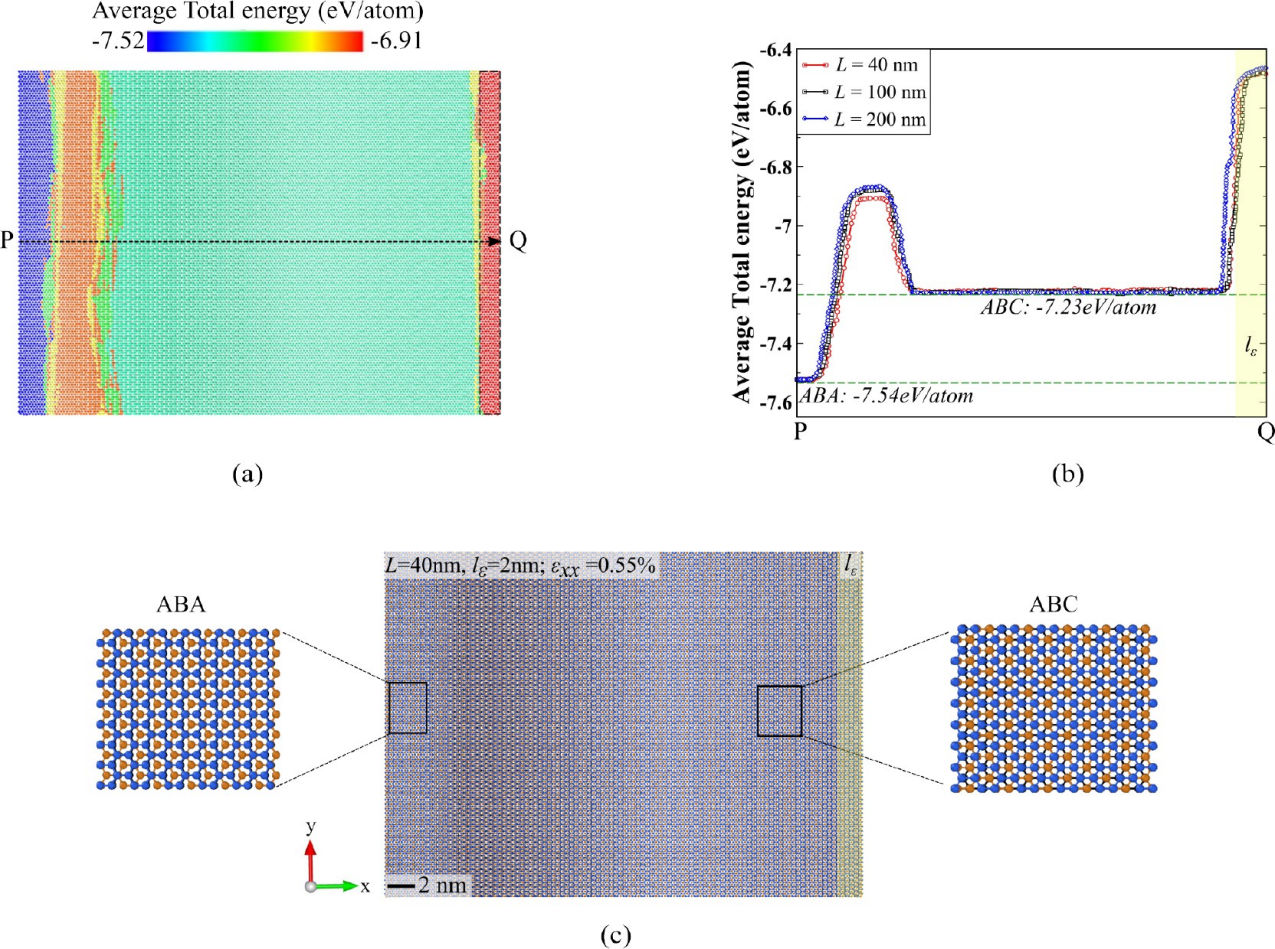
Energy distribution and stacking identification. (a) Contour plot showing the total energy distribution across the loaded flake, averaged with respect to its nearest neighbor for ε_*xx*_ = 0.55% (slippage strain) configuration. The black dotted box indicates the tab region. (b) The energy profile at slippage along the shown path PQ. The unchanged ABA stacking on the unstrained end exhibits the lowest energy (−7.54 eV/atom), while the transformed ABC stacking region shows a homogeneous energy distribution (−7.23 eV/atom). The intermediate region displays a local energy maximum, with the highest energy confined to the tab region. (c) Atomic snapshot at 0.55% strain during the slip process, highlighting the uniformly strained tab (*l*_ε_ = 2 nm; 5% of the flake length) in yellow. Two zoomed-in snapshots show regions with ABC-TLG (right) and unchanged ABA-TLG (left) configurations.

We examine structural changes in the flake by identifying local stacking order reconfiguration at atomic length scales. Differentiating between various stackings in TLG poses a challenge due to the similarities in the interlayer distance and lattice parameters (see Table S1). Nevertheless, the MS model allows the evaluation of variations in energy contributors that can distinguish between different stackings. Force fields excel at exploring large interfaces that provide a unique capability to study local properties across extensive length scales. These simulations extract atomic-level information like force, energy, and strain for each atom in the system. In contrast, ab initio DFT calculations, known for obtaining more accurate structural properties are computationally intensive and usually limited to smaller systems. We follow a similar approach to our previous study to monitor total energy (TE) across the length of the flake and use this measure to identify stacking configuration of atoms.^62^ We average TE for each atom with its nearest bonded neighbors to smooth energy magnitudes per atom as shown in Figure 3(a). Plotting the average TE per atom along a defined path “PQ” (including the tab region) reveals two distinct regions with constant TE/atom magnitudes, separated by two energy maxima (Figure 3(b) for *L* = 40 nm). The high-energy peak at the tab region is due to rigid boundary conditions applied to the atoms of this region. We observe uniform TE quantities spanning a large region concentrated in the middle of the flake as well as another confined region at the free end of the flake. These uniform energy profiles are consistent in magnitude with the energy of pristine ABC and ABA stacking as shown in Figure 3(b) (also see Table S1) signifying the presence of distinct crystal structures localized within the system. The high TE region observed toward the free end of the flake is associated with an intermediate stacking region that facilitates the transition of atomic arrangements from ABA to ABC domains.

The real-space atomic snapshots in Figure 3(c) corroborate the observation drawn from the top layer energy profile. The flake edge on the free side that has the lowest energy value (−7.54 eV/atom) shows the presence of ABA stacking. Conversely, the middle area that relaxes to a local energy minimum (−7.23 eV/atom) consists of the ABC stacked crystal structure. Based on this analysis, we classify atoms with an energy value of −7.54 eV/atom as ABA stacking and −7.23 eV/atom as ABC stacked TLG. As shown in Table 1, the lattice parameters obtained from the atomic configurations in Figure 3(c) closely align with the lattice parameters of pristine ABA and ABC structures calculated using DFT, consistent with earlier studies.^10,17,63^ These lattice constants and angles reflect the formation of distinct crystal structures after slippage: hexagonal (ABA) and rhombohedral (ABC). Atoms with other energy values, such as those corresponding to high-energy peaks are classified as an intermediate stacking phase that is neither ABC nor ABA. It is important to note that the classification method is demonstrated using the TE/atom contour plot of the flake at ε_*xx*_ = 0.55% essentially because this is where the rearrangement is detected. Furthermore, the energy distribution across the flake remains highly similar for different flake widths, allowing the use of the same energy thresholds for stacking identification regardless of flake size (Tables S1 and S2). However, we exclude the tab (*l*_ε_) region from the outcome of this identification scheme due to its fixed and unrelaxed atomic configuration. Consequently, this stacking identification method effectively delineates three distinct regions within the flake: ABA-TLG, ABC-TLG, and an intermediate/mixed stacking phase.

**Table 1:** Comparison of Lattice Parameters (*a*_stacking_) and Total Energy of Pristine and Strain-Engineered TLG Domains (at ε_*xx*_ = 0.55%)

	Lattice constant (Å)	Lattice angles (deg)			
Structure	*a*	α	β	γ	Crystal system
Pristine ABA	2.463	90	90	120	Hexagonal
ABA	2.459	90	90	120	Hexagonal
Pristine ABC	2.496	60	60	60	Rhombohedral
ABC	2.503	60	60	60	Rhombohedral

#### 3.2.2 ABA to ABC-TLG Structural Transition

**Figure 4:**
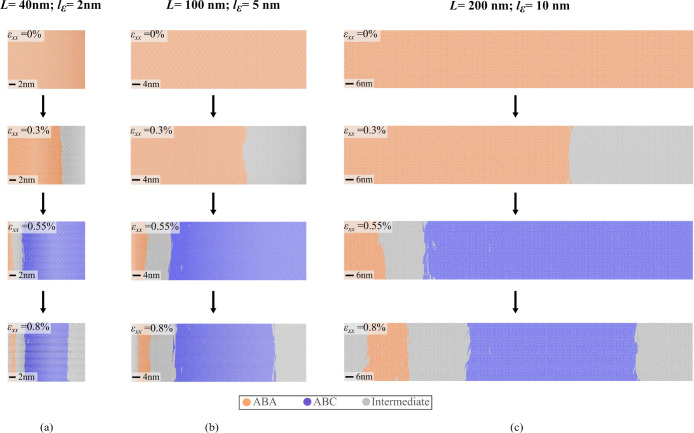
Strain-induced stacking order change. The stacking order transitions and evolution of ABC domains for different flake dimensions with a tab width corresponding to 5% of the flake width: (a) *L* = 40 nm, *l*_ε_ = 2 nm, (b) *L* = 100 nm, *l*_ε_ = 5 nm, and (c) *L* = 200 nm, *l*_ε_ = 10 nm. The behavior of stacking order change remains consistent across all the considered flake dimensions.

We employ our stacking classification scheme on atomic snapshots at different strain magnitudes to visualize the strain-induced transition from ABA to ABC stacking (Figure 4). As we start deforming the initial ABA flake by straining the tab region, a gradual depletion of pure ABA stacking becomes evident primarily in the vicinity of the tab. This observation aligns with the top layer strain propagation profile prior to slippage and exhibits an early decay while traversing the edges of the flake (Figure 2(c)). Consequently, the formation of the intermediate stacking order is observed to grow as strain continues to increase until slip occurs leading to the formation of a large domain with ABC-TLG stacking configuration (Figure 4).

Upon slippage (ε_*xx*_ = 0.55%), the enhanced strain transmission in the top layer transforms a significant portion of the flake into the locally stable ABC configuration. However, the free edge of the flake retains its ABA stacking configuration. This behavior is due to the limited strain experienced by this region since it is away from the loading edge as observed in the strain profile shown in Figure 2(b), demonstrating a reduction in strain values from point Q to point P. This phenomenon leads to the formation of an intermediate high-energy region that separates the transitioned ABC stacking region from the retained ABA stacking order as seen in Figure 3(b) and Figure 4. As a result, we observe three distinct regions in the loaded flake after slippage, i.e., a large domain of the transitioned ABC configuration, the unchanged ABA region at the free end of *l*_ε_ and the intermediate soliton area (gray region) in between (Figure 4(a)–(c)). Deforming stacked 2D materials by stretching in-plane bond interactions incurs a higher total energy cost compared to displacing in-plane bonded atoms without altering their covalent bonds.^58^ Hence, continued loading postslip facilitates gradual sliding of the top layer that causes changes in the structure but with a gradual energy cost (Figure 2(c)). We observe further transitions in the stacking configurations when loading continues beyond slippage due to this sliding behavior. The ABC-TLG domains gradually shrink starting from the vicinity of the tab region and transform into an intermediate stacking. Moreover, we also observe a shift of ABA domains toward the loading direction with the appearance of intermediate stacking at the free end. These stacking transitions are demonstrated for ε_*xx*_ = 0.8% in Figure 4 underscoring the intricate dynamics involved in reordering of atoms.

This stacking order transformation and evolution of the ABC domain is consistently observed across all considered flake dimensions, highlighting the robustness of our method (Figure 4(a)–(c)). The most significant conversion from ABA to ABC-TLG stacking occurs at the critical strain of ε_*xx*_ = 0.55%. We further calculate the volume fraction of each stacking as a function of applied strain to quantitatively assess this stacking order change (Figure S2). The volume fraction of each region (*x*) is determined by *V F*_*x*_ = *V*_*x*_/*V*_total_, where *V*_*x*_ represents the volume occupied by atoms in either the ABA or ABC stacking and *V*_total_ is the total volume of the entire flake in real space. Our findings reveal transformation in the unstrained ABA flake, with approximately 80% of the configuration transitioning to ABC upon slippage followed by a gradual depletion (Figure S2(a,b)). It is also important to note that this volume fraction change remains nearly consistent across various widths of the strained tab and for different flake sizes (Figure S2(c,d)). This indicates that the stacking transition behavior is consistent regardless of the flake length scale and is not influenced by the width of the enforced rigid boundary conditions along the tab region. This observation can be attributed to the similarity in the rigid boundary conditions and strain propagation characteristics within the flake.

### 3.3 Stacking Transition under Varied Loading Orientations

**Figure 5:**
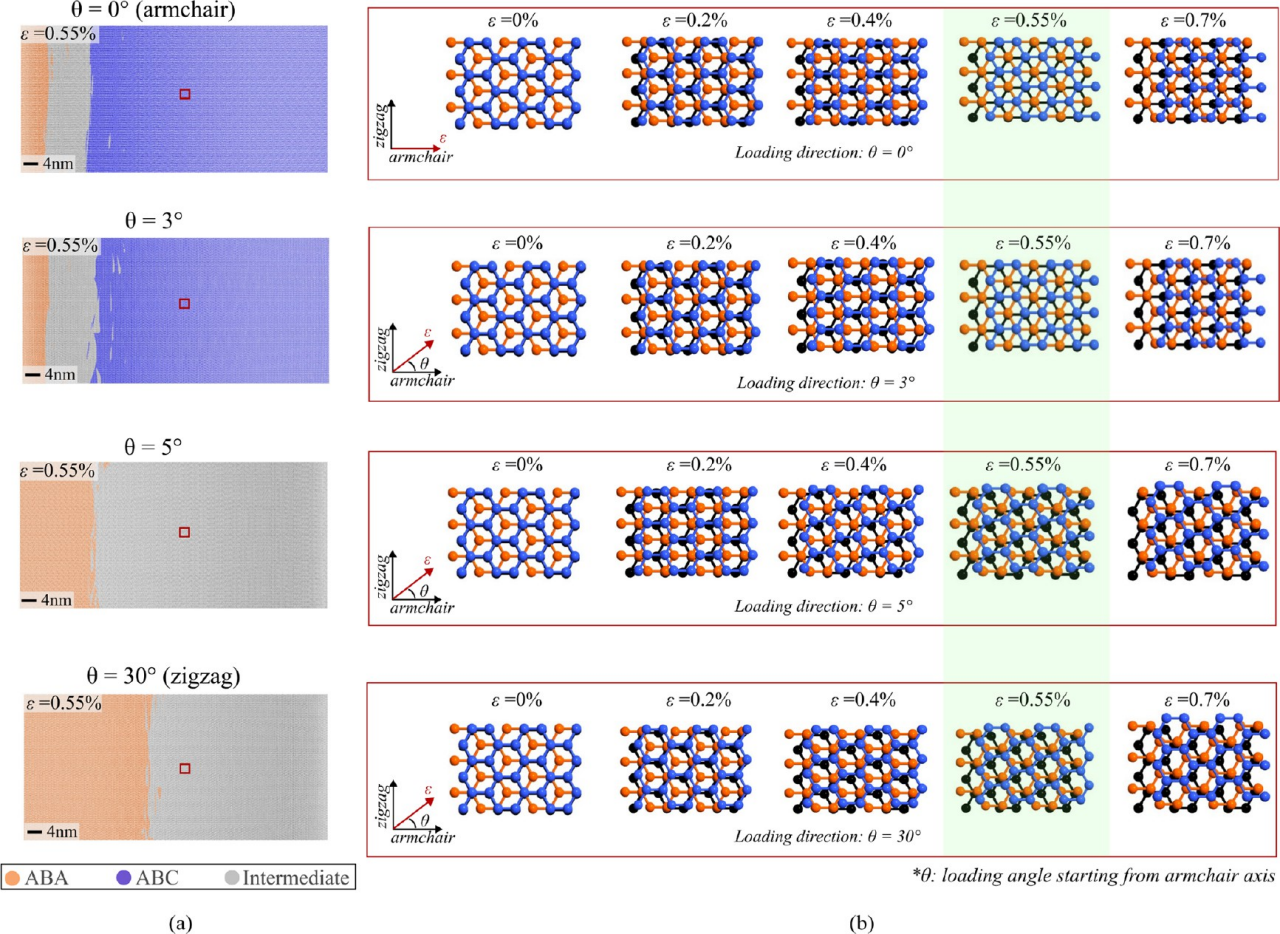
Stacking change mechanism under varied loading directions. (a) Atomic configurations at slippage strain (ε_*xx*_ = 0.55%) for different loading orientations (θ = 0°, 3°, 5°, and 30°) with identified stacking regions. Loading angle θ represents the deviation from the perfect armchair (θ = 0°) axis. (b) Close-up snapshots of the atomic structure within the small area outlined by the red box in (a), illustrating the stacking change mechanism starting from the unstrained configuration. The loading orientation is indicated by the ε direction in the coordinate axes, that also shows the perfect armchair and zigzag axes. The atomic snapshots at slippage for each loading direction are shaded in light green.

We further studied the influence of loading axis orientation on the stacking transition as illustrated in Figure 5. It is known that zigzag and armchair axes differ by a 30° orientation, and we have denoted the perfect armchair axis as θ = 0° orientation. Our analysis shows that with some minute deviation from the perfect armchair direction (shown for θ = 3° case), a similar stacking transition behavior is observed. This results in the majority of the flake transforming from ABA to ABC stacking upon slippage. However, when there is a significant deviation as observed for θ = 5° loading orientation, the flake does not relax into the perfect ABC configuration upon slippage. Instead, some portions of it form an intermediate configuration, while other portions retain the ABA stacking order (Figure 5(a)).

A closer examination of a locally selected atomic configuration further helps in understanding the direction-dependent stacking transition. The fundamental reason behind this anisotropic stacking change lies in the geometric arrangement and subsequent deformation induced by the applied load. A detailed inspection in Figure 5(b) reveals that perfect ABC formation is achieved when the C atoms in the top “A” layer subtly shift to the next lattice site along the armchair direction. This shift precisely situates them at the center of the hexagonal ring formed by the C atoms in the middle “B” layer and aligns them exactly on top of the C atoms in the bottom “A” layer. Excessive deviation from the ideal armchair axis results in the top layer C atoms shifting in an oblique direction, hindering the formation of ABC stacking. Given that the deviation from the armchair axis is not too substantial in the case of θ = 5° loading, its initial energy pathway likely involves searching for the ABC local energy minimum. However, the atomic configuration in this case demands higher energy compensation to transition toward ABC stacking than to maintain its current configuration. To better understand the mechanism of abrupt shift in stacking behavior between θ = 3° and 5°, we examined the impact of different loading directions on atomic displacements near the slippage point (Figure S4). As we slightly deviate from the perfect armchair (*x*-axis), the primary displacement component is along armchair axis, including a minor component along the perpendicular (*y*-axis). Continuous loading results in positive displacement magnitudes for both components until slippage. However, the configuration for θ = 3° load upon slippage relaxes by having negative *y*-axis displacement. In contrast, θ = 5° loading maintains positive *y*-axis even after slippage. This behavior shows that for small deviation from armchair axis, the strained flake favors relaxing back to ABA with a small negative *y*-axis displacement, whereas the same behavior becomes energetically unfavorable for increased deviation.

For perfect zigzag loading (θ = 30°), the top layer C atoms cannot effectively rearrange themselves by sliding to the adjacent favorable lattice sites because the atomic arrangement does not allow it. Consequently, upon slippage, the flake relaxes into an intermediate arrangement and further sliding leads to the top layer C atoms returning to the initial ABA stacking lattice. Furthermore, when strain starts to decay in the other end of the top layer, loading along the perfect zigzag axis results in larger remaining ABA domains compared to the θ = 5° loading case. This outcome is primarily because zigzag loading induces symmetrical deformation along a perfect axis that causes atoms to be displaced along energetically favorable lattice sites. The atoms rearrange themselves and relax back to ABA stacking with the decay of strain, as the energy compensation allows them to release strain rather than maintain the strained state. Conversely, the atomic configuration in the θ = 5° loading case does not favor such relaxation. Due to asymmetrical deformation, even with strain decay, the atoms persist in the strained state until the point at which the energy required to revert to the ABA stacking is lower than maintaining the strained state. These findings underscore the highly anisotropic nature of the stacking change detected through our strain-based approach. The loading orientation plays a pivotal role in influencing the structural alignment of the atoms and the specific deformation imposed on the lattice, thereby impacting the stacking transition. The demonstrated mechanism can be utilized to better understand the techniques that resulted in unstable and nonuniform ABC stacking order.

### 3.4 Stability of ABC Configuration

The stability of different stacking configurations in TLG is influenced by several factors including interlayer interactions, lattice symmetry, and energy minimization.^64^ ABA stacking is known to be a more stable configuration^65,66^ compared to ABC stacking due to the favorable alignment of the carbon atoms and the strong interlayer interactions between them.^3,67^ The ABC stacking obtained by inducing the stacking order change is considered a metastable configuration due to its higher TE/atom. This means that although the system can temporarily adopt the ABC configuration, it is energetically less favorable and the system might be prone to reverting back to the stable ABA configuration upon removing the mechanical constraint.

**Figure 6:**
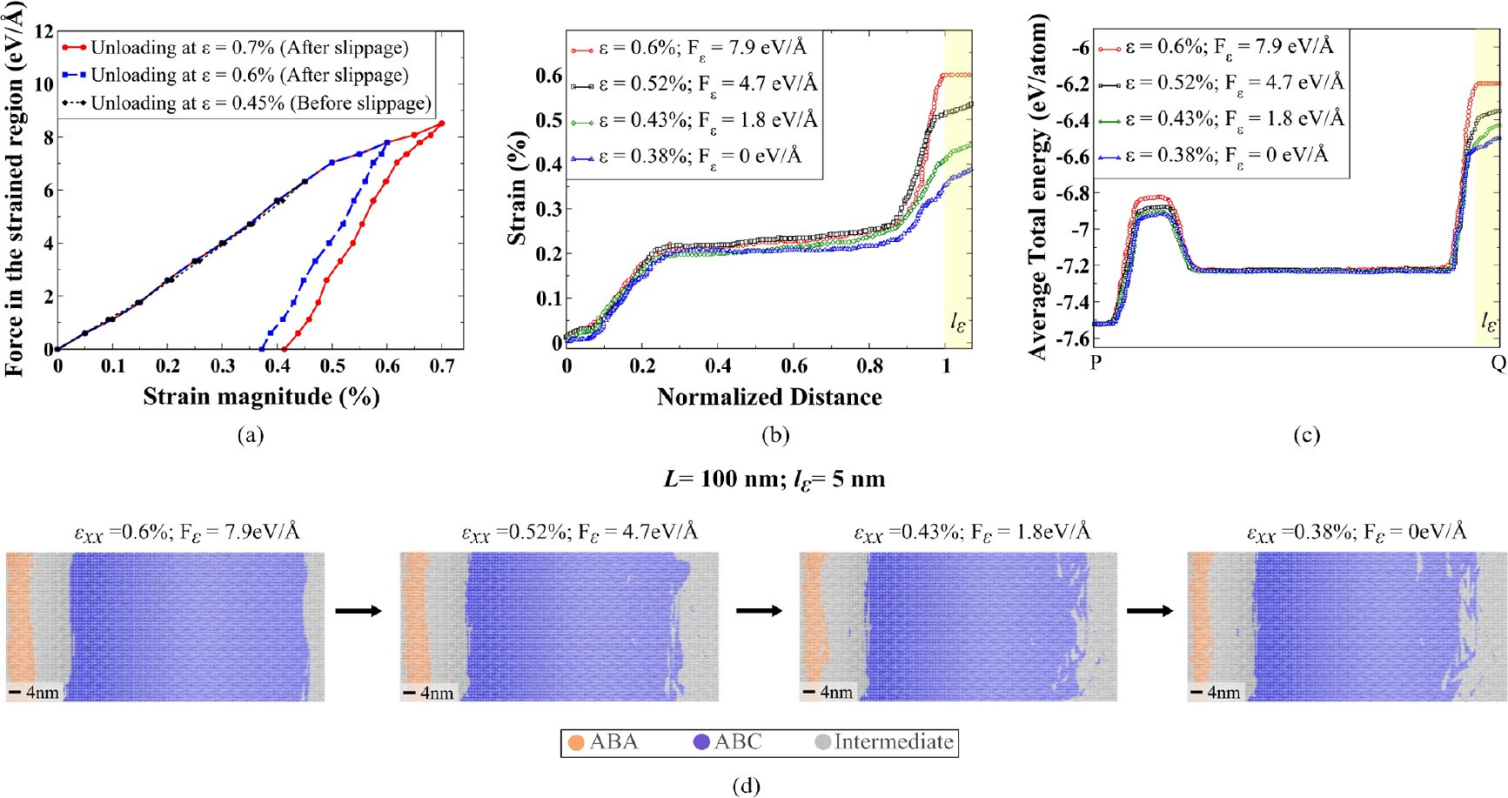
Stability of ABC configuration upon unloading. (a) Force versus strain plot during unloading, showing elastic loading behavior before slippage and plastic deformation after slippage. The residual strain magnitudes reported in the tab region during unloading represent the average strain, calculated by averaging the strain per atom. (b) Strain profile of the top layer during unloading at different intervals. The legend indicates the average strain in the tab for each corresponding unloading stage. (c) Local energy distribution along the path PQ (Figure 3(a)) reveals an energy barrier that hinders significant structural changes in the ABC-TLG region. (d) Atomic snapshots during unloading, showcasing stable ABC structure formation with minimal changes after load reduction.

By unloading the strained structure, we examine if the ABC stacking configuration remains stable without external forces. This allows us to understand its capability to maintain ABC atomic arrangement when the stressor is removed. Our results show that upon unloading from a strained configuration above slippage strain (at 0.6% and 0.7%), the tab region has some residual strain indicative of plastic deformation (Figure 6 (a)). An average residual plastic strain magnitude of 0.38% remains in the tab region when we reduce the force to zero while unloading from 0.6% and similarly, 0.4% strain remains while unloading from 0.7%. However, unloading before slippage strain (at 0.45% as shown in Figure 6) is completely elastic. The emergence of plastic deformation and the observed residual strain in the *l*_ε_ region upon unloading can be attributed to the structural rearrangements and energy landscapes present in the system. When the external force is removed during unloading, the configurational resistance provided by the atoms adjacent to and outside the tab region prevents a complete relaxation of the material back to its original state. The presence of residual strain even when the force is completely reduced to zero demonstrates the existence of some energy landscapes that hinder a complete return to the original ABA configuration.

We again analyze the total energy quantity (TE/atom) across the flake for the atomic configurations while unloading to identify the structural changes during the process. Plotting TE/atom along the same path PQ shown in Figure 3(a) for different stages during unloading from ε_*xx*_ = 0.6% reveals a similar energy profile where we have a constant energy region for both ABA (at the free edge, near P) and ABC stacking (in the middle), with a peak in between corresponding to the intermediate configuration and a plateaued maximum for the tab region (Figure 6(c)). This maximum energy peak in *l*_ε_ region indicates irreversible structural changes and an inability to fully recover the original configuration even in the absence of external force. The ABC-TLG region remains encapsulated between the two surrounding energy peaks indicating that the system is energetically trapped in this configuration. This suggests that the energy landscape and the structural arrangement of the ABC stacking are the reason for the stability of ABC and hinder the system from transitioning into other high-energy states upon unloading (Figure 6(c)).

Additionally, the observation that the strain profile of the top layer during unloading exhibits similar propagation behavior as observed at slippage (ε_*xx*_ = 0.55%, Figure 2(b)) implies that the transformed ABC stacking preserves its atomic configuration (Figure 6(b)). It shows that the structural changes induced by the ABA to ABC transformation, particularly in the top layer, are stable and resistant to relaxation or reverting back to the ABA configuration. The energy profile shown in Figure 6(c) indicates that deformation is more concentrated in the intermediate stacked domains and the level of strain does not change considerably in the ABC region during unloading. This is a further indication of the stability of ABC domains, clearly supporting its stability arguments. These observations are evident in atomic snapshots during the unloading process (Figure 6(d)). While some deformations to intermediate configurations are observed at the edge near the tab region when starting from the ε_*xx*_ = 0.6% state, the majority of the ABC configuration and the atomic structure at the free end remain intact. Hence, our stability analysis demonstrates the effectiveness of our method in preserving the ABA to ABC stacking order change.

### 3.5 Experimental Characterizations

**Figure 7:**
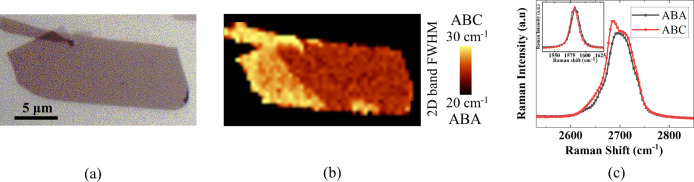
Exfoliation and Raman spectra of TLG flakes. (a) Exfoliated trilayer graphene flake on SiO_2_ substrate. (b) Raman map of the 2D band fwhm to distinguish ABA and ABC stacking orders. (c) Raman spectra of 2D band for ABA and ABC-TLG, the inset shows the G band Raman spectra.

We further performed experimental measurements using Raman to study the stacking order change. The exfoliated TLG flake typically exhibits ABA stacking order that is more stable compared to the ABC stacking. However, our experimental sample shown in Figure 7(a) demonstrates the coexistence of both stacking orders within the same exfoliated TLG flake. To distinguish between these stacking configurations, Figure 7(b) displays the Raman map of the fwhm of the 2D band in this specific sample. The Raman intensity ratio of G to 2D band is used to accurately identify the thickness of the graphene samples.^68^ Furthermore, Figure 7(c) visually illustrates the distinct difference in shape and fwhm of the 2D band between ABA and ABC stacking orders that provides an effective means for their identification.^69^ The inset of Figure 7(c) presents the G band spectra of the ABA and ABC structures where a difference of ∼2 cm^–1^ is observed in the G band frequency. Based on the Raman spectroscopic mapping on our exfoliated flake, we chose the samples with full ABA stacking order for the experiments. Additionally, this selection makes the analysis of strain and stacking order changes simpler by not taking the effect of domain walls between the regions (solitons) into account. Also, it must be noted that the pre-encapsulation optical images have enhanced contrast in order to accurately identify the thickness and ensure no wrinkles or bubbles have formed, while the postencapsulation optical images have lower contrast to keep both covered and uncovered parts visible.

#### 3.5.1 Strain Engineering with Evaporated Stressed Thin Films

The process-induced strain engineering technique is employed to induce layer slippage and alter the stacking order in graphene through controlled strain application. This method has been implemented in industrial CMOS technology since the 90 nm technology node to enhance electron or hole mobility in transistor channels.^70^ Previously, we have demonstrated this technique in exfoliated 2D materials using evaporated stressed thin films.^55^ We showed that strain type (tensile/compressive) and magnitude can be controlled through thin-film material selection and film force (= film stress × film thickness). In this method, compressive thin films (MgO, SiO_2_) expand to relieve the stress and induce tensile strain, while films with tensile stress like MgF_2_ induce compressive strain by contracting to alleviate stress. We also have innovated a method for precise strain control within 2D materials, enabling deterministic design for uniaxial or biaxial strain and alignment with crystal axes. Achieved through lithographically patterned thin film stressors,^54^ akin to stripe geometry stressors in silicon technology inducing uniaxial strains at edges,^71^ these induce controlled uniaxial strain aligned with desired crystal axes. These methods are nondamaging, offering robust time and temperature stability.^72^ Moreover, we observed natural incomplete out-of-plane strain transfer (heterostrain) in 2D structures through this method due to weak vdW coupling.^46,55,73^ This enables precise layer-by-layer strain engineering in few-layer 2D materials. In graphene, under 15% of the top layer strain is transmitted to the second layer, with no further propagation.^73^ In this section, we show how this unique feature of graphene enables us to significantly strain the top layer of a TLG and cause layer slippage with strategic manipulation of process-induced strain.

**Figure 8:**
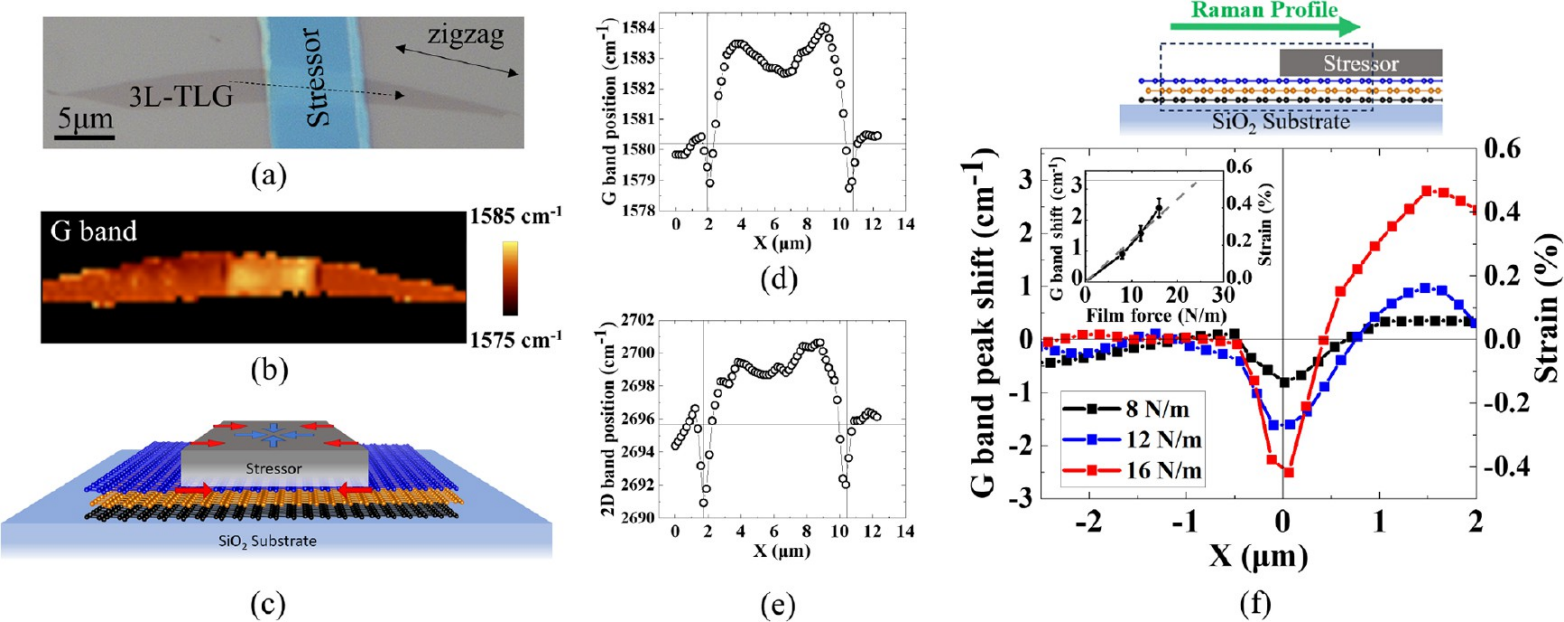
Raman characterization of TLG flake with deposited stressor. (a) ABA-TLG flake coated with the stressor with a stripe geometry to create uniaxial strain at the edges. (b) Raman map of the G band Raman mode. Darker regions at the edge indicate tensile strain while the center shows compressive strain. (c) The geometry of strain distribution created with the striped stressors. (d) and (e) Raman peak position of G band and 2D band across the dashed line. (f) G band Raman profile after separating the doping effect and estimated strain magnitude across the stripe edge with different film forces. The inset presents the G band peak shift and strain magnitude at the edge of the stripe.

Figure 8(a) depicts the optical image of the device geometry implemented in this study. The figure shows a trilayer graphene flake initially in ABA stacking order, coated with a thin film stressor with a stripe geometry. We observed earlier in our simulation results that the uniaxial strain-induced stacking order change occurs along the armchair axis of the TLG lattice. Previous research indicates that taller and straight edges in multilayer flakes can serve as identifiers of the zigzag axis while edges with corrugated atomic structures and terracing may indicate the armchair axis.^74,75^ A systematic analysis is presented in the Supplementary Section IV showing the approach we used to detect the trilayer flake edges. We then strategically pattern the stressors to induce uniaxial strain along the armchair direction by identifying the zigzag axis in the exfoliated graphene flakes (shown in Figure 8(a)). The Raman map of the G band for the corresponding device in Figure 8(b) reveals downward shifts at the edges and upward shifts at the stripe center, that can be respectively ascribed to tensile and compressive strains. The distribution and type of strains in the TLG flake generated from the stripe stressors can be observed in Figure 8(c). Due to the tendency of the stressor to contract, the free edges create unidirectional forces toward the inside of the stripe and perpendicular to the stressor edge creating uniaxial tensile strain at the edges. Meanwhile, the central region experiences compressive forces resulting in biaxial compressive strain. This unique strain distribution is similar to what has been observed in 2D and 3D systems with stripe stressors.^54,71^

Figure 8(d),(e) presents the G band and 2D band Raman mode profiles along the dashed line in Figure 8(a). Through the analysis of the 2D band frequency versus G band frequency (Figure S8), we have separated the effect of doping from strain on the Raman responses of the samples to accurately analyze the strain magnitude.^76^ The strain-induced G band peak shift across the edge of the stripe stressors with different film forces is shown in Figure 8(f). We note that after eliminating the effect of charge density on the Raman response, the observed shifts in G band indicate the presence of compressive strain at the center of the stripe. However, the downshift of the G band at the edges indicates a narrow region along the edges with high tensile strain (as presented in Figure 8(b)). Employing a stressor film with higher film force results in a larger Raman peak shift in both the edge and the center of the stripe indicating larger strain. Previous work reports the edge-induced tensile strain to be located within a very narrow region.^71^ Hence considering the Raman laser spot size, measuring the exact Raman peak profile and strain magnitude in this region is challenging. The presented Raman peak profiles are the convolution of the actual Raman peak profile with the Gaussian distribution of the Raman laser intensity (2σ = ∼1 μm).^71^ Additionally, as shown previously by our group, the observed Raman peak shift in TLG samples is an average of the Raman spectra collected from every single layer with different strain magnitudes (100%, 15%, and 0% from top to bottom).^73^

Considering these approximations and using the translation factor to convert the G band peak shift to uniaxial strain magnitude in graphene reported before,^77^ an estimate of the strain magnitude in the top layer is included in Figure 8(f). The inset of Figure 8(f) presents the maximum Raman peak shift at the edge and the corresponding strain magnitude for different film forces. The linear fit shown by the dashed line suggests a film force of ∼25 N/m can create 0.55% strain that is enough for the layer slippage in graphene. Consistent with previous findings in three-dimensional systems featuring an evaporated stressor in a stripe geometry, the uniaxial strain induced by the edges extends beyond the boundaries of the stressor.^71^ This behavior aligns with the modeling performed in the simulation section. The film force of the stressor in this particular device is 16 N/m with ∼0.4% strain in the top layer of TLG that falls below the magnitude of strain required to observe a change in stacking order.^58^ Therefore, a typical strain distribution is observed in this sample. Following the change in stacking order resulting from layer slippage, strain solitons are formed in the regions with uniaxial strain resulting in strain relaxation at the edges.

#### 3.5.2 Strain-Induced Layer Slippage and Stacking Order Changes

**Figure 9:**
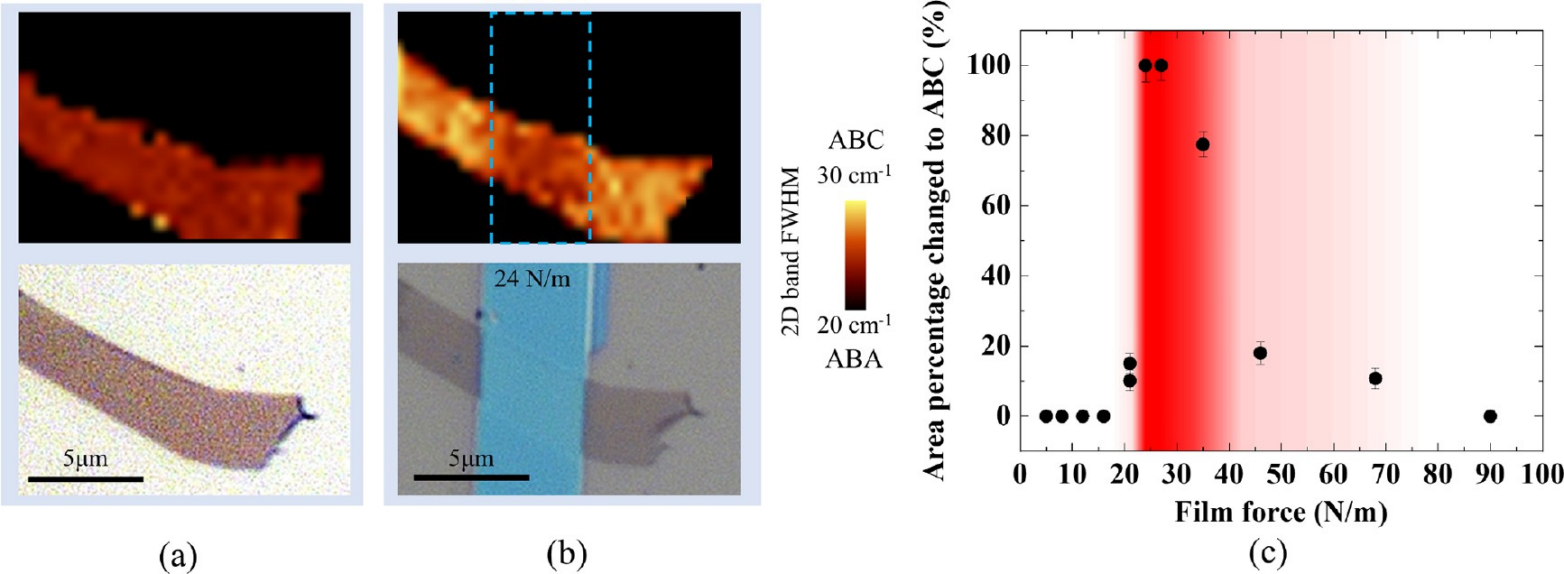
Optical images demonstrating the stacking order change. (a) ABA-TLG before deposition of the stripe stressor. (b) ABA to ABC stacking order changes in TLG after deposition of strip stressor with 24 N/m film force. (c) The area percentage of TLG changed from ABA to ABC after stressor deposition (stressor-covered part not considered). The red-colored area presents the film force boundaries where maximal ABA to ABC changes are observed. The error bars are calculated based on the areas of any residual ABA and ABC regions that fall below the Raman resolution.

The initial ABA sample (Figure 9(a)) undergoes a stacking transition to ABC upon depositing the stressor with a film force of 24 N/m (Figure 9(b)). In this device, and with reference to Figure 8(f) data, we estimate a uniaxial edge strain along the armchair axis that surpasses the critical graphene strain threshold of 0.55%. However, this strain is confined to the narrow-edge regions. The Raman map of the 2D band fwhm highlights areas outside the stripe in the trilayer sample undergoing a stacking order change to ABC. This change impacts the shape and fwhm of the 2D band in Raman spectra and offers insights into strain-induced structural modifications in the ABA-TLG sample. We further examine the effect of stressor film force on stacking order changes. The percentage of ABA-to-ABC transition area relative to the stressor film force is illustrated in Figure 9(c). The comprehensive analysis and methodology employed to determine the change in ABA to ABC area fractions from Raman optical images are presented in Supplementary Section VI. Below ∼20 N/m, the strain is not enough to induce layer slippage (<0.5%), yielding no stacking change, whereas beyond 20 N/m, the top layer when pulled toward the stressor edge begins slipping on the second layer and forms ABC regions. At a film force of 24 N/m, slippage occurs across the entire layer leading to the complete switching of ABA domains to ABC. It must be noted that the Raman laser spot size is ∼1 μm and Raman spectra in the maps are collected with 0.5 μm steps. Hence, any mixed ABA/ABC regions or remaining ABA regions with areas below 1.5 × 1.5 μm^2^ cannot be resolved. In this case, the collected Raman spectrum is an average of the ABA and ABC regions and their areas cannot be precisely resolved within this limit. Thus, while we report 100% ABA-to-ABC change in Figure 9(c), the presented error bars denote the percentage of areas with fwhm between the ABA and ABC stacking order. This reflects the potential existence of remaining ABA or mixed regions, as predicted by our simulation results (Figure 4), that is not fully resolved by Raman spectroscopy. By further increasing the film force beyond 24 N/m the area of ABC regions starts to shrink, similar to what we observe in our simulations (Figure 4). Raman mappings of the devices included in Figure 9(c) are presented in Figure S7. These results prove that using the proposed lithographically patterned evaporated stressors, large enough uniaxial strain can be generated along the armchair axis, in the top layer of the TLG flakes leading to layer slippage and a new lattice configuration and stacking order.

The proposed method enables a versatile ABA to ABC stacking order change in TLG, without stringent limitations on flake length. Stacking order alterations have been successfully observed/hlup to 10 μm away from the edge of the stressor (illustrated in Figure S7). We further extended our investigation to explore the stacking order change behavior when subjecting the flake edge to strain from two sides (see Supplementary Section VII). In this setup, the stressor induces tensile strain on both sides of the top layer without any free boundary conditions. Similar to the single-sided straining, we observe an ABA to ABC transition during slippage strain. The stacking order change is visible through Raman optical images in Figure S11 and atomistic simulation snapshots of this model, as showcased in Figures S12 and S13. These findings establish the reliability of our approach, utilizing patterned evaporated thin film stressors, for engineering stacking orders and phases in various 2D materials through induced stacking or phase transitions using uniaxial strain.

In order to characterize and gain insights into the formed local stacking at atomic scale, we further conducted theoretical Raman analysis using phonon dispersion calculations based on DFT simulations. Starting from the strained structure obtained at a strain magnitude of 0.55% determined through MS models, we isolate a primitive unit cell of the ABC stacked region and the remaining ABA region (see Supplementary Section III). Additionally, we considered regions adjacent to and away from the strained tab to examine the 2D band data derived from the phonon frequencies at the *K* point in the Brillouin zone.^78^ Our observations revealed a distinct difference in the 2D peaks between the two stacking configurations, aligned with our experimental data and findings reported in the literature (Table S3). Also, we observe a close alignment of the 2D band data for these domains when compared to their pristine structure. This alignment remained consistent for different flake lengths, suggesting that the stacking order change leads to similar crystal structures independent of the flake size. Hence, our finding serves as strong evidence of the successful formation of stable ABC domains in the strained TLG flake. The theoretical Raman analysis based on phonon dispersion calculations not only confirms the presence of ABC stacking but also enriches our understanding of the strain-induced structural modifications and phonon behavior in the material. Besides Raman, various characterization techniques can further enhance our analysis of the formed ABC domains. Transport measurements offer valuable insights into the electronic properties of the ABC regions that are possible by deposition of metallic stressor contacts with unique geometries. In addition to these studies, several other techniques are applicable to characterize ABC stacking in TBG. Methods such as scanning tunneling microscopy (STM) and transmission electron microscopy (TEM) can be employed to image the TLG surface, while angle-resolved photoemission spectroscopy (ARPES) allows for the direct measurement of the band structure of the ABC domains. We have reserved these characterization techniques for future studies. We anticipate that the combination of these techniques will yield a comprehensive understanding of the ABC stacking regions in TBG, which is crucial for developing TBG-based devices with the desired characteristics.

## 4 Conclusion

In this work, we studied the mechanism of stacking order change in trilayer graphene (TLG) and examined the underlying interfacial process responsible for transition of Bernal (ABA) to rhombohedral (ABC) domains. Through a combination of atomistic simulations and experimental techniques, we gained valuable insights into the interfacial mechanism governing the stacking transformation in TLG under strain. Our findings revealed that by applying a striped stressor to a localized area of the top layer in the ABA-TLG flake, interlayer slippage is induced, resulting in the formation of stable ABC-TLG domains. The strain-induced transition was characterized by a critical strain magnitude of 0.55%. Beyond this threshold, the strain distribution underwent significant changes, resulting in interlayer sliding and a subsequent change in stacking order. We monitored the evolution of this transition by analyzing the atomic configurations using total energy per atom quantity and observed distinct local domains within the TLG flake, including ABA-TLG, ABC-TLG, and an intermediate/mixed stacking phase. Importantly, this stacking order transformation from ABA to ABC was consistently observed across different flake widths demonstrating the robustness of our proposed technique. We examined the anisotropic nature of stacking change by studying its mechanism under varying loading directions. Transition to ABC domains was only observed when loaded along a perfect and slightly deviated armchair axis.

Furthermore, we investigated the stability of the obtained ABC-TLG stacking by removing the mechanical constraint. Results indicated the presence of ABC-TLG domains even without external stimuli, and this was experimentally verified by observing the ABC domains after stressor removal. The presence of energy barriers between stacking configurations trapped the system energetically in the ABC configuration, thus preserving this stacking order. To characterize the obtained structural transition, we utilized experimental Raman spectroscopy and DFT simulations, focusing on the 2D peak data. The analysis of these data provided further confirmation of the successful stacking order change and validated the structural stability of the ABC-TLG domains. Hence, our work addresses the challenges associated with obtaining and preserving stable ABC stacking in TLG and opens up new possibilities for the fabrication and characterization of ABC graphene. Moreover, it demonstrates a universal mechanism that drives the stacking change under the influence of mechanical strain. This advancement holds significant potential for the development of advanced electronic and optoelectronic devices in TLG-based systems.

## Data Availability

The data supporting the findings of this study are available from the corresponding author upon reasonable request.

## Supporting Information Available

Additional analysis of structural properties of strain-engineered TLG domains compared to pristine configuration; analysis of stacking order change for different lengths of tab region; phonon dispersion spectra of the obtained ABA- and ABC-TLG domains; additional figures for volume fraction of ABA to ABC transformation; atomic displacement contours for two different loading orientations. description of detection of flake edges; calculation of local domains area fraction from Raman images; description of robustness of stacking order changes with striped stressors; analysis of stacking order change when strained from both edges of the flake. optical image of TLG flakes with initial ABA stacking orders; Images showing the TLG flakes before and after depositing the stripe stressors; atomic snapshots and energy profiles of stacking transformation when strained from both ends (PDF)
