# Supplementary material for: Uniaxial Strain-Induced Stacking Order Change in Trilayer Graphene
Source: ACS Appl Mater Interfaces. 2024 Jan 31;16(6):8169–83. doi: 10.1021/acsami.3c19101 (PMC10875650; doi:10.1021/acsami.3c19101)
Supplement: Supplementary file 1 — am3c19101_si_001.pdf [file am3c19101_si_001.pdf]

Supporting Information

Uniaxial Strain-Induced Stacking Order Change in Trilayer Graphene

Aditya Dey*,[1, a)] Ahmad Azizimanesh,[2] Stephen M. Wu,[2, b)] and Hesam Askari[1]

[1)] *Department of Mechanical Engineering, University of Rochester, New York*

[2)] *Department of Electrical and Computer Engineering, University of Rochester, Rochester, New York*

[a)] Electronic mail: adey2@ur.rochester.edu

[b)] Department of Physics and Astronomy, University of Rochester, Rochester, New York

I. STRUCTURAL ANALYSIS OF STRAIN-ENGINEERED TLG DOMAINS COMPARED TO PRISTINE CONFIGURATION

We conducted a comprehensive analysis of the crystal structures of various TLG stacking configurations resulting from strain engineering. Our investigation focused on understanding the structural properties of the domains under the slippage strain condition ($\epsilon_{xx} = 0.55\%$) where a maximum transformation from ABA to ABC stacking occurred during the slippage process. As evident, we observed the formation of three distinct regions within the flake during slippage strain: ABC-TLG along the edge of the strained region, unchanged ABA-TLG at the other end, and some intermediate stacking between the two. To gain insights into the full spectrum of structural transitions, we selected different regions within the domain areas and calculated their lattice parameters. For the transformed ABC-TLG domain, we identified two specific spots: $ABC_\epsilon - I$, located close to the strained region (l_ϵ), and $ABC_\epsilon - II$, which was farther away from l_ϵ. Additionally, we selected a single region (ABA_ϵ) from the unchanged ABA-TLG domain, as it constituted a relatively smaller volume fraction (Figure S1).

To analyze the lattice parameters, we extracted the atomic coordinates from these selected regions and obtained the average bond lengths (l_{avg}) for each configuration. From this data, we derived the corresponding in-plane lattice constants as $a_{stacking} = \sqrt{3} l_{avg}$ [1–4]. To further investigate the stability of these configurations, we conducted energy minimization simulations using Density Functional Theory (DFT) and obtained their relaxed lattice constants and total energy. In addition to the strain-engineered TLG domains, we also performed similar simulations for pristine ABA- and ABC-TLG structures. By comparing the results, we observed that the obtained properties for both stacking configurations aligned remarkably well with the ones obtained via strain engineering. This finding suggests that during interlayer slippage, as the structure relaxes to local minima, the resulting distinctive TLG regions are not entirely novel structures but rather bear similarities to the pristine TLG stackings. Moreover, we noticed a high degree of spatial uniformity within the ABC domains, as both ABC-I and ABC-II regions exhibited closely aligned lattice constants and energy parameters. This spatial uniformity throughout the domain area indicates a consistent structural behavior in the strained TLG. The close resemblance of these domains to the pristine TLG stackings and the spatial uniformity within the ABC regions indicate a

controlled and consistent structural transformation during the slippage process. We showed this analysis for all the flake widths considered (Table S1), as well as for different lengths of the strained region for the L=100nm case.

II. STACKING ORDER CHANGE FOR DIFFERENT LENGTHS OF STRAINED REGION

As discussed earlier, to induce the ABA to ABC transformation, we applied localized stressors on the top layer of the initial ABA-stacked TLG, which resulted in strain propagating outward from the stressor-deposited region. The length of this strained area (l_ϵ) at the flake edges played a crucial role in driving the stacking order change. To compre-hensively understand the dependency of the stacking order change on the length of the l_ϵ region, we conducted systematic experiments with various l_ϵ percentages at the edge of the ABA top layer. We explored a range of percentages, going up to 10%, to assess the impact of deformation within these regions. Specifically, we varied the percentage of the strained region in steps of 2.5%, including 2.5%, 5%, 7.5%, and 10%, while keeping the variation con-sistent across all three considered flake lengths. Maintaining a uniform deformation length scale was crucial for accurate analysis, so we ensured that the free length of the flake outside the strained region remained constant for each configuration. For instance, in Fig. S1, with a flake length (L) of 100 nm and a l_ϵ of 5 nm (5% of the flake length), we extended the total length of the flake to 105 nm. This adjustment guaranteed that the region experienc-ing strain remained consistent at 100 nm. Surprisingly, our observations revealed that the length of the strained region (l_ϵ) did not significantly influence the stacking order transition. As depicted in Figure S2, the volume fraction change (for L=100nm case) from ABA to ABC at slippage strain exhibited remarkable similarity across different l_ϵ magnitudes, hovering at around 80% for all cases. These results indicate that the slip phenomena and underlying strain propagation during the stacking transition are not dependent on the specific length scale of the strained region. Instead, they follow a constant phenomenon irrespective of the l_ϵ region length. The consistency of the stacking order transition regardless of the length of the strained region opens up interesting possibilities for strain engineering in TLG. This suggests that the underlying physics governing the stacking transformation remains uniform and predictable, which could be advantageous for tailored applications and device design

based on strain engineering in trilayer graphene.

III. PHONON DISPERSION SPECTRA OF THE OBTAINED ABA- AND ABC-TLG DOMAINS

We performed DFT simulations to calculate the phonon dispersion spectra of the obtained ABA- and ABC-TLG domains at $\epsilon_{xx} = 0.55\%$ and extract their 2D Raman band data. We employed self-consistent density functional perturbation theory (DFPT) to simulate the phonon dispersion spectra of all the structures [5–7]. DFPT is a powerful theoretical framework that enables us to investigate the vibrational properties of materials by perturbing their electron density. To initiate the phonon calculations, we first computed the dynamical matrices for each TBG structure. These matrices were computed on an adequate q-point grid, ensuring that the phonon wave vectors were properly sampled to obtain accurate and reliable results [8, 9]. The inter-atomic force constants obtained from the dynamical matrices were then used to calculate the phonon dispersion. To achieve this, we employed Fourier interpolation techniques to extract the phonon frequencies at various wave vectors throughout the Brillouin zone [10, 11]. We used the unit lattice cell parameters $a_{stacking}$ of different regions (Figure S1) to compute their phonon spectra.

Figure S3 displays the phonon spectrum obtained for the ABC domain (Region $ABC_\epsilon - II$) of a sizable L=100nm flake along the Γ-K-M-Γ high symmetry path in the Brillouin zone. As per the methodology adopted by Popov et al. [12], we utilized twice the magnitude of the transverse optical (TO) frequency at the K point to extract the 2D band data. This unique 2D band is intimately linked with an intriguing intervalley scattering process involving two phonons at the K point in the material's reciprocal lattice [12–14]. It originates from an inelastic scattering event, arising when an incident photon interacts with these two phonons. The phonons participating in this process are transverse optical (TO) phonons, which bring about vibrations in the lattice, marked by atomic displacements perpendicular to the phonon's propagation direction [15, 16]. In Table S2, we have compiled the calculated 2D band frequencies for various regions of the TLG domains. Notably, we observe remarkable alignment of the 2D band data for these domains when compared to the pristine structure. Moreover, a clear distinction emerges in the frequencies between ABA (2701 cm^{-1}) and ABC-TLG (2665 cm^{-1}) domains, underscoring the characteristic differences between these

stacking orders. An intriguing observation is that the 2D peak data aligns very closely for different flake widths, indicating that the stacking order change results in similar crystal structures, independent of the flake size.

IV. DETECTION OF TRILAYER GRAPHENE EDGES (ZIGZAG AND ARMCHAIR)

To identify the zigzag and armchair axes of graphene samples in this work we have employed statistical analysis of the graphene edges. Initially, we make sure to keep all the exfoliated graphene flakes on each chip oriented in the same direction. This is done during the tape exfoliation process in which the graphene flakes are thinned by an unused piece of scotch tape while they are kept in the original orientation. This way all the resulting flakes are oriented along the axes of the original single-crystalline graphene. It should be noted that exfoliating the graphene flakes by attaching two pieces of scotch tape with graphene crystals, twisted relative to each other results in the graphene flakes having various orientations and should be prohibited to keep all the graphene flakes oriented in the same direction. We then extract the length and angle of the graphene flakes' edges throughout the whole substrate using optical images as presented in Figure S5a. The length vs angle of the graphene edges is then used to calculate the cumulative edge length as a function of the angle that is presented in Figure S5b. This figure presents the cumulative edge lengths sharing the same angle across multiple flakes on the chip and shows that zigzag edges (at 30°, 90°, and 150°) exhibit higher cumulative lengths compared to the armchair edges expected at 30° difference. In previous studies, HRTEM imaging of graphene edges shows that straight edges in multilayer graphene may serve as an indicator of the zigzag axis, whereas edges exhibiting corrugated atomic structures and terracing may indicate the presence of the armchair axis [17, 18] which potentially yields longer zigzag edges compared to armchair edges. As suggested by the mentioned work and many others [17, 19], 2D materials with hexagonal structures have a higher tendency to cleave along the zigzag axis and typically long straight edges in these materials identify the zigzag axis.

To facilitate the process of axis identification in this work, a Python code based on Canny edge detector [20] is written and used to identify edges from the contrast difference in the optical images and consequently, calculate the length and the angle of the edges

and calculate the cumulative length of edges at each specific edge angle. Figure S5(c)-(e) presents the optical image of multiple graphene flakes fed to the edge detector Python code and green lines mark the identified edges by the code. Figure S5(e) presents the polar plot of cumulative edge length as a function of edge angle throughout the whole chip. The zigzag axes show significantly larger cumulative lengths as shown by the red arrows at 30°, 90°, and 150° axes.in Fig. S5b. The work by Guo et. al. [21] reports the statistical analysis of edge count as a function of angle regardless of the edge length. Our observations show that cumulative edge length rather than count could be an accurate identifier of the graphene axis. Additionally, the results by Guo et. al. report that cleavage along one of the zigzag axes has nearly double the likelihood of occurrence compared to the other axes in graphene which are similar to our observations presented in Figure S5(c)-(e). To confirm the relative direction of the stressor to the axes of graphene flakes a similar analysis is performed on each single substrate presented in this work. Figure S6 presents the graphene axis identification analysis from four separate chips and as expected the direction of uniaxial strain is confirmed to be along the armchair axis of the graphene flake.

V. ROBUSTNESS OF STACKING ORDER CHANGES WITH STRIPE STRESSORS

Figure 7 presents the 2D band FWHM Raman maps and optical images of TLG graphene flakes before and after stressor deposition. In figures 7(a) and (b) no stacking order changes are observed. In figure 7(c) with film force of 21 N/m, a small area around the right edge has changed to ABC. Figure 7(d) shows a device that consists of trilayer and stripe stressor with an optimal film force of 24 N/m. The Raman map of 2D band FWHM of this flake shows full ABA to ABC stacking order changes in this sample. Figure 7(e) presents another example of the uniaxial strain-induced stacking order changes in a relatively tall (>20 μm) TLG flake. This figure supports our theoretical hypothesis from the main text that the stacking order change in TLG is caused by a uniform top layer slippage in the TLG and is not limited by the flake length as we observe stacking order changes up to 10um away from the stressor's edge. Figures 7(e)-(h) show the effect of increasing the film force of the stressor. Further film force generates enough strain and causes a second slippage which switches the newly formed ABC regions back to ABA stacking order. Figure 8 presents the

effect of strain and doping from the evaporated stressor on the TLG samples. The 2D band vs. G band of graphene shows different movement trajectories as presented here.

VI. CALCULATION OF ABA TO ABC AREA FRACTION CHANGE FROM RAMAN OPTICAL IMAGES

The resolution of the Raman mapping has an impact on the calculated area of ABC and ABA regions. This uncertainty leads to error in considering a Raman spectra ABA or ABC and the area of the newly formed ABC regions. In order to take the effect of unresolved ABA/ABC regions on the Raman spectra and accurate area calculation, we extract the histogram distribution of the 2D band FWHM from the Raman maps. Figure S9(b) presents the 2D band FWHM histogram of samples shown in Figure S9(a). As expected, the FWHM has Gaussian distributions with centers around 24 cm^{-1} and 28 cm^{-1} for the 2D band FWHM of ABA and ABC trilayer graphene, respectively. Any spot with 2D band FWHM between these values could potentially be a region with mixed ABA/ABC stacking order areas smaller than the Raman spot size since the Raman spot collects an average of Raman spectra from both regions.

The Y axis of the histogram plot is the number of single Raman scans from the Raman map, which means the area under the histogram plot directly translates to the area of the sample. This enables the accurate calculation of the ABA and ABC regions area, as presented in Figure S9(c). To accurately identify the ABA and ABC spots from the histogram plot without any arbitrary judgments, the histogram is fitted with Gaussian functions as presented in Figure S9(c). The two Gaussian functions identify the range of 2D band FWHM of each spectrum to be considered ABA or ABC trilayer graphene. If a region with mixed ABA/ABC stacking order exists, we expect the 2D band FWHM to fall in the range where the two Gaussian functions overlap and we cannot certainly assign it to ABA or ABC.

To account for the Raman resolution on the calculation of area and area percentage changes, error bars are calculated and added to Fig 9c in the main text. These error bars have been computed based on the areas of any spots from the Raman map with indiscernible FWHM values. Figure S10 presents the 2D band FWHM distribution extracted from the Raman map of the same devices from Figure S7 with 4 different film forces. Using the FWHM distribution information, we can accurately calculate the ABC area percentage

from the Gaussian fittings of these distributions. Additionally, error bars can be estimated and added to the calculated areas to make them more accurate. Using the new approach of calculating the ABC area percentage and errors originating from the Raman spot size, Figure 9c of the main text is presented.

VII. STACKING ORDER CHANGES BY STRAINING BOTH SIDES OF ARMCHAIR EDGE

We extended our investigation to explore the stacking change behavior when subjecting the flake edge to strain from both ends, eliminating any free boundary conditions. In this scenario, the stressor is positioned to induce tensile strain on both sides of the top layer. The atomistic model for an L=200 nm sample and the corresponding experimental setup is depicted in Figure S11. Similar to the single-sided strain case we observe both in-plane and out-of-plane strain propagation, which becomes more pronounced with increasing strain magnitude. Analyzing the top layer strain profile reveals a monotonic decrease in in-plane strain propagation from both flake ends, ultimately leading to slippage at 0.55%. As slippage occurs, the strain around the flake center reaches a plateau marked by two maxima and a central dip (Figure S12(a)). The examination of total energy of the system at the point of slippage aligns with the top-layer strain profile. A consistent energy region corresponds to the formation of ABC domains, while a small dip signifies the unchanged ABA region (Figure S12(b)).

The evolution of ABC stacking through bidirectional straining becomes evident as we analyze stacking-identified snapshots at different strain levels (Figure S12(c)). Initial strain prompts the depletion of ABA regions from the edges and at the point of slippage, the top layer shifts in the direction of the applied strain that instigates the change in stacking order. This directional slipping behavior generates strain-concentrated soliton regions at the center, which aid the slippage by maintaining ABA domains within the middle to balance the total energy. The stability of the formed ABC domains is tested upon unloading the flake, revealing an energy landscape that, similar to the one-sided straining mechanism confines the ABC domains within high-energy states and prevents their return to ABA (Figure S13). The experimental configuration involving striped stressors for bidirectional straining yields analogous outcomes. An ABA to ABC transition occurs at a film force of 24 N/m, mirroring

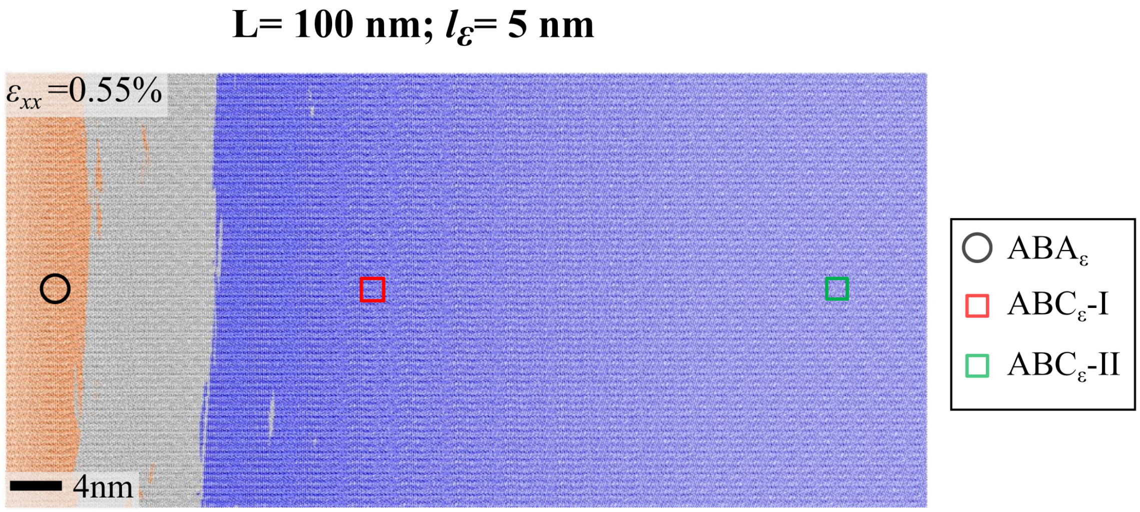

FIGURE S1: Visualization of distinct regions within the obtained trilayer graphene (TLG) domains under slippage strain ($\epsilon_{xx} = 0.55\%$). The depicted illustration corresponds to a TLG flake with a length (L) of 100 nm and a characteristic length of the slippage r egion (l_ϵ) of 5 nm.

TABLE S1: Comparison of in-plane lattice constant (a) and interlayer distance (d) of pristine ABA trilayer graphene obtained from different methods.

Method	a (Å)	d (Å)
PBE-GGA, vdW-DFT-D2 (this work)	2.463	3.344
PBE-GGA, vdW-DF [22]	2.464	3.342
LDA [23]	2.45	3.35
PBE-GGA, vdW-DFT-D3 [24]	2.461	3.3
Experiment [25]	2.458	3.337

the observed phenomena.

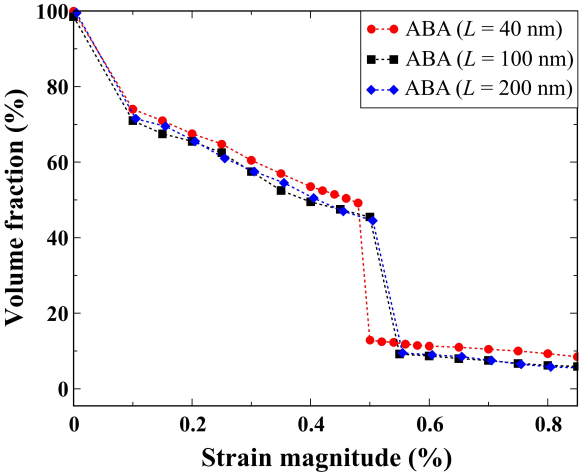

(a)

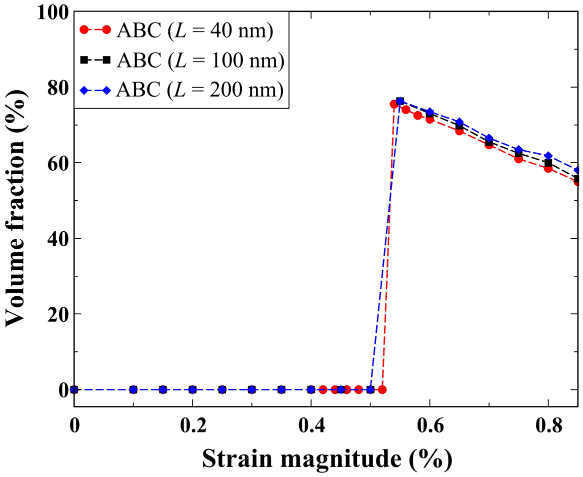

(b)

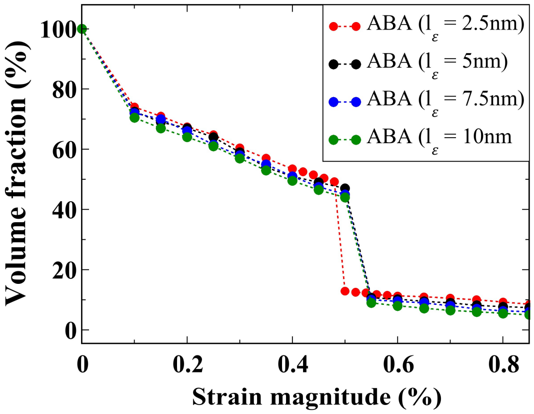

(c)

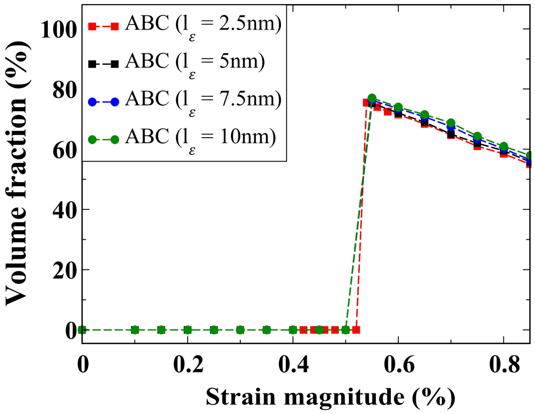

(d)

FIGURE S2: The change in stacking configuration is examined by analyzing the volume fraction change of (a, c) ABA and (b,d) ABC domains for various flake widths (a-b) and various lengths of strained region for L=100 nm case (c-d). The results demonstrate that 80% of the initial ABA-TLG undergoes a transformation to the ABC structure during the slippage process. This volumetric change remains consistent across various flake lengths and for any l_ϵ magnitude.

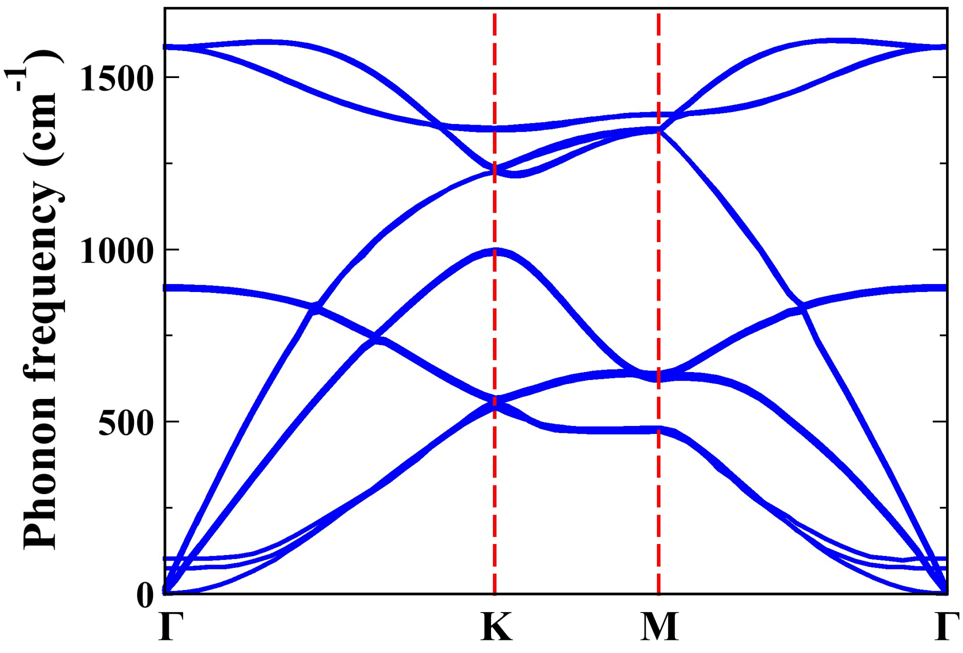

FIGURE S3: Phonon dispersion spectra of ABC-TLG domains obtained at ϵ_{xx} = 0.55% for L=100nm case.

TABLE S2: Comparison of in-plane bond length (l_{avg}), lattice parameter ($a_{stacking}$) and total energy of pristine and strain-engineered TLG domains (at ϵ_{xx} = 0.55%) for all the flake widths (L_1 =40nm, L_2 =100nm, and L_3 =200nm)

	l_{avg} (Å)			$a_{stacking}$ (Å)			Total energy (eV/atom)		
Structure	L_1	L_2	L_3	L_1	L_2	L_3	L_1	L_2	L_3
Pristine ABA	1.422	1.422	1.422	2.463	2.463	2.463	-7.54	-7.54	-7.54
ABA_ϵ	1.419	1.421	1.420	2.462	2.459	2.458	-7.52	-7.53	-7.51
Pristine ABC	1.441	1.441	1.441	2.496	2.496	2.496	-7.23	-7.23	-7.23
$ABC_\epsilon - I$	1.447	1.445	1.444	2.508	2.503	2.505	-7.21	-7.25	-7.23
$ABC_\epsilon - II$	1.445	1.444	1.446	2.504	2.499	2.506	-7.22	-7.24	-7.23

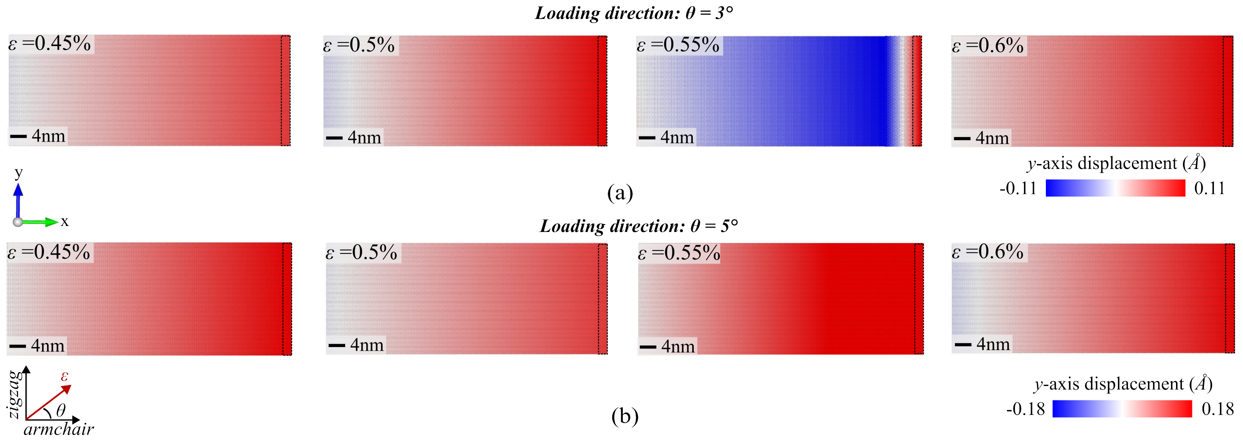

FIGURE S4: Atomic displacement contour plots for θ = 3° and θ = 5° loading directions along y-axis. The displacement magnitudes for both orientations are shown by the respective color bars.

TABLE S3: Comparison of in-place bond length (l_{avg}), lattice parameter ($a_{stacking}$) and total energy of pristine and strain-engineered TLG domains (at $\epsilon_{xx} = 0.55\%$) for L = 100nm flake and different widths of the strained region

Structure	l_{avg} (Å)		$a_{stacking}$ (Å)		Total energy (eV/atom)	
	l_ϵ=5	l_ϵ=10	l_ϵ=5	l_ϵ=10	l_ϵ=5	l_ϵ=10
Pristine ABA	1.422	1.422	2.463	2.463	-7.55	-7.55
ABA_ϵ	1.419	1.417	2.459	2.455	-7.53	-7.53
Pristine ABC	1.441	1.441	2.496	2.496	-7.28	-7.28
$ABC_\epsilon - I$	1.447	1.445	2.503	2.508	-7.24	-7.21
$ABC_\epsilon - II$	1.445	1.448	2.504	2.509	-7.22	-7.26

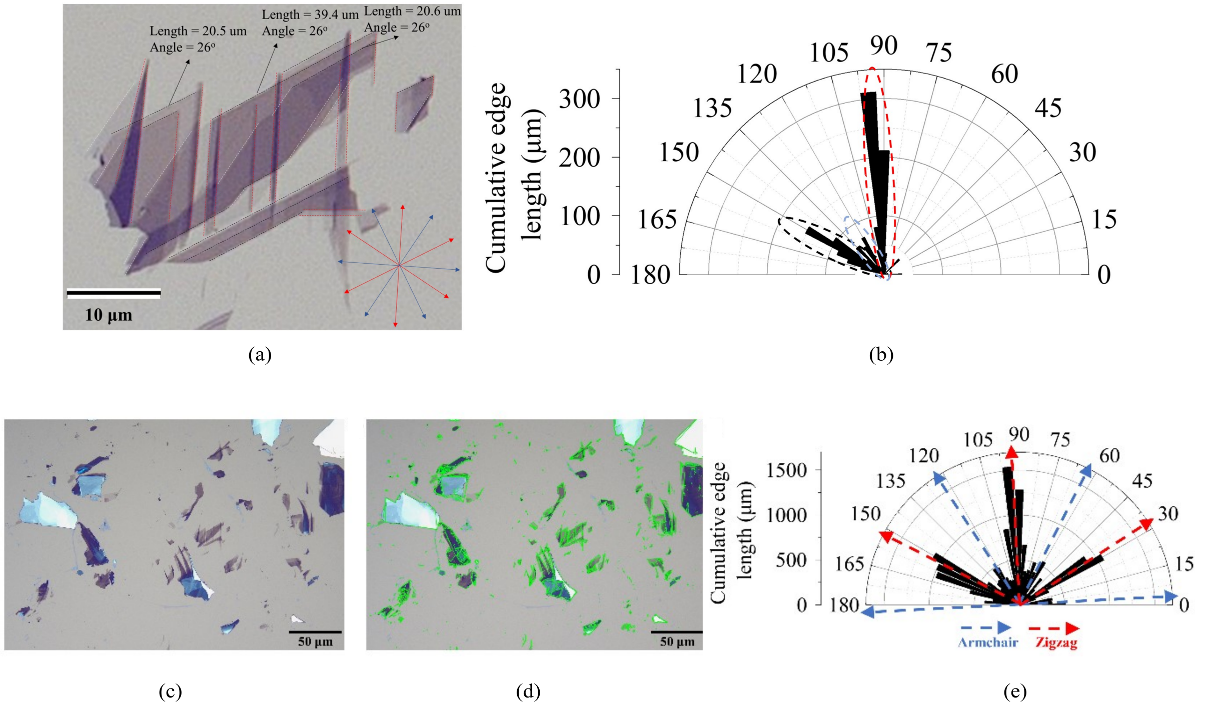

FIGURE S5: (a) Optical image of a graphene flake used to analyze the length and angle of the edges. (b) Cumulative length of graphene edges as a function of the edge angle. (c) Optical image of multiple graphene flakes used to analyze the length and angle of the edges. (d) Identified edges by the Python code. (e) Cumulative length of graphene edges as a function of the edge angle.

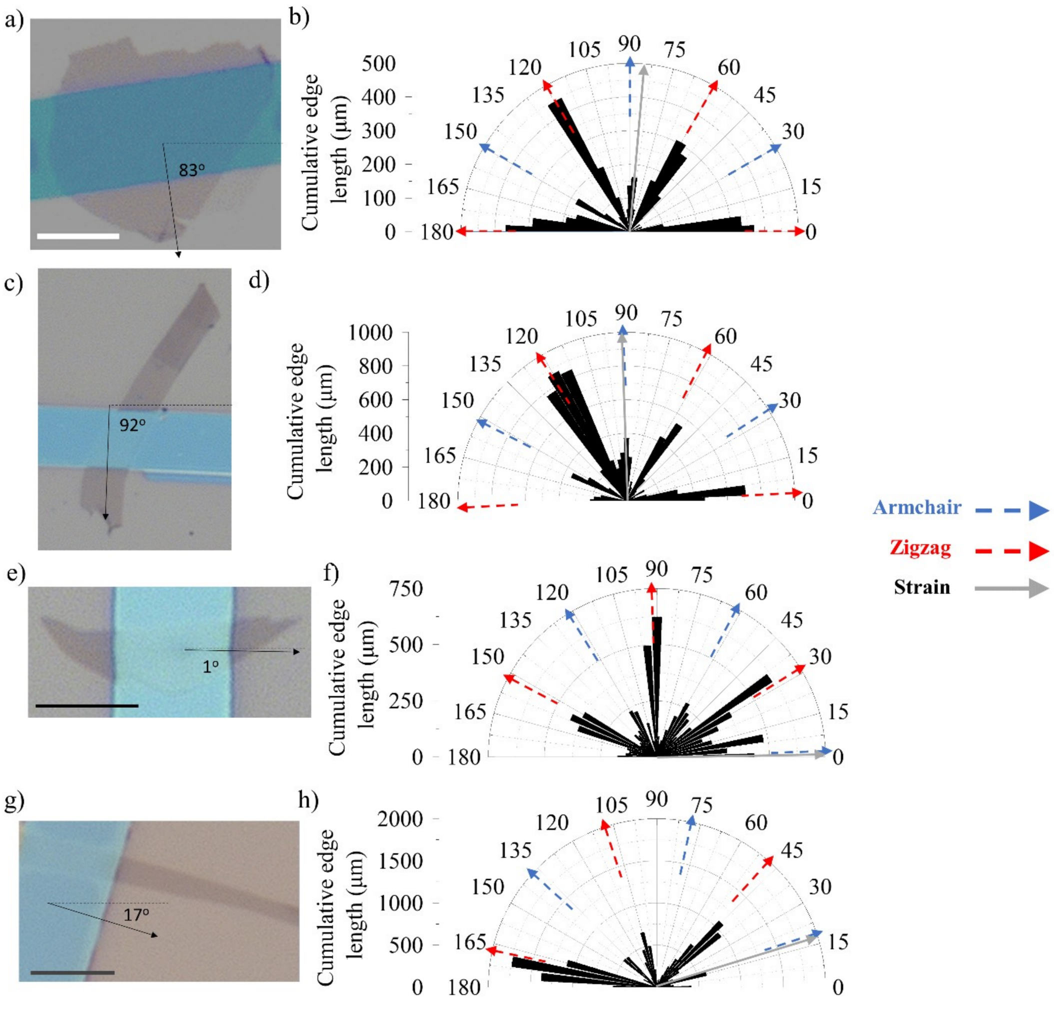

FIG. 6: a) and b) Optical image and Crystal axes of the graphene sample with 8 Nm-1. c) and d) Optical image and Crystal axes of the graphene sample with 24 Nm-1. e) and f) Optical image and Crystal axes of the graphene sample with 35 Nm-1. g) and h) Optical image and Crystal axes of the graphene sample with 27 N/m.

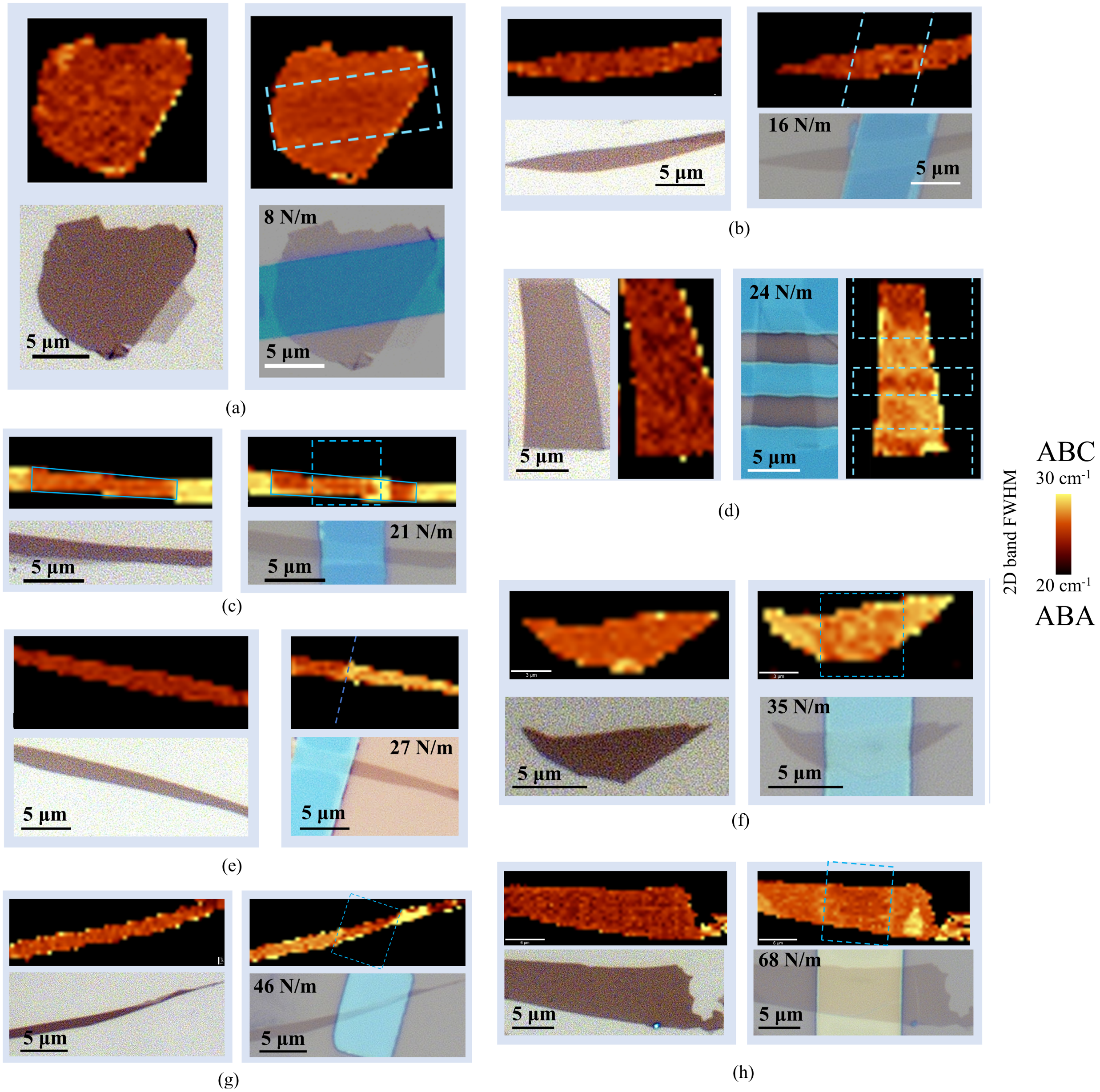

FIGURE S7: 2D band FWHM Raman map and optical image of TLG samples before and after stressor deposition with film forces of (a) 8 N/m, (b) 16 N/m, (c) 21 N/m, (d) 24 N/m, (e) 27 N/m, (f) 35 N/m, (g) 46 N/m, and (a) 68 N/m. ABA to ABC Stacking order changes are observed with film forces above 21 N/m.

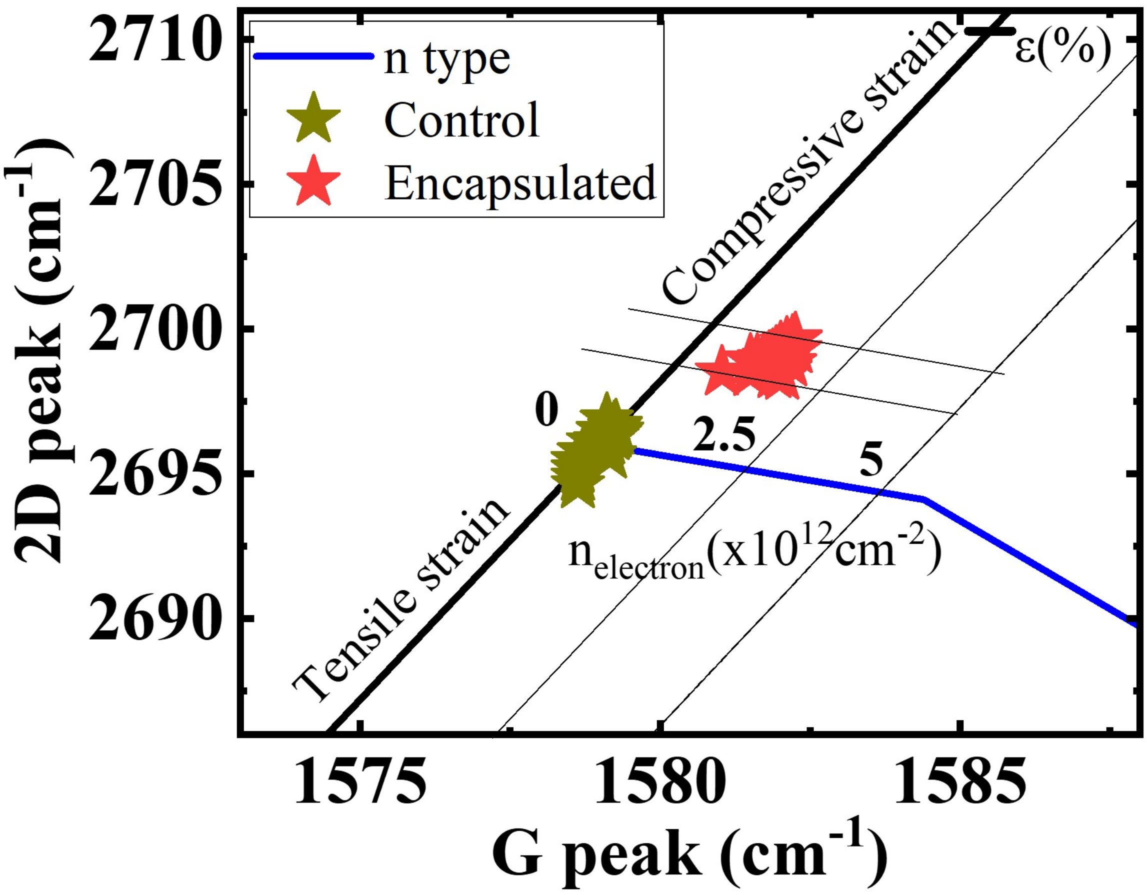

FIGURE S8: 2D band vs G band plot of a TLG graphene utilized to separate the strain effect

TABLE S4: Comparison of 2D band Raman frequency of pristine and the transformed ABA to ABC-TLG domains at $\epsilon xx = 0.55\%$ for all the considered flake widths

	2D band frequency (cm^{-1})		
Structure	L=40nm	L=100nm	L=200nm
Pristine ABA	2698	2698	2698
ABA_ϵ	2701	2703	2700
Pristine ABC	2667	2667	2667
$ABC_\epsilon - I$	2662	2665	2668
$ABC_\epsilon - II$	2664	2668	2666

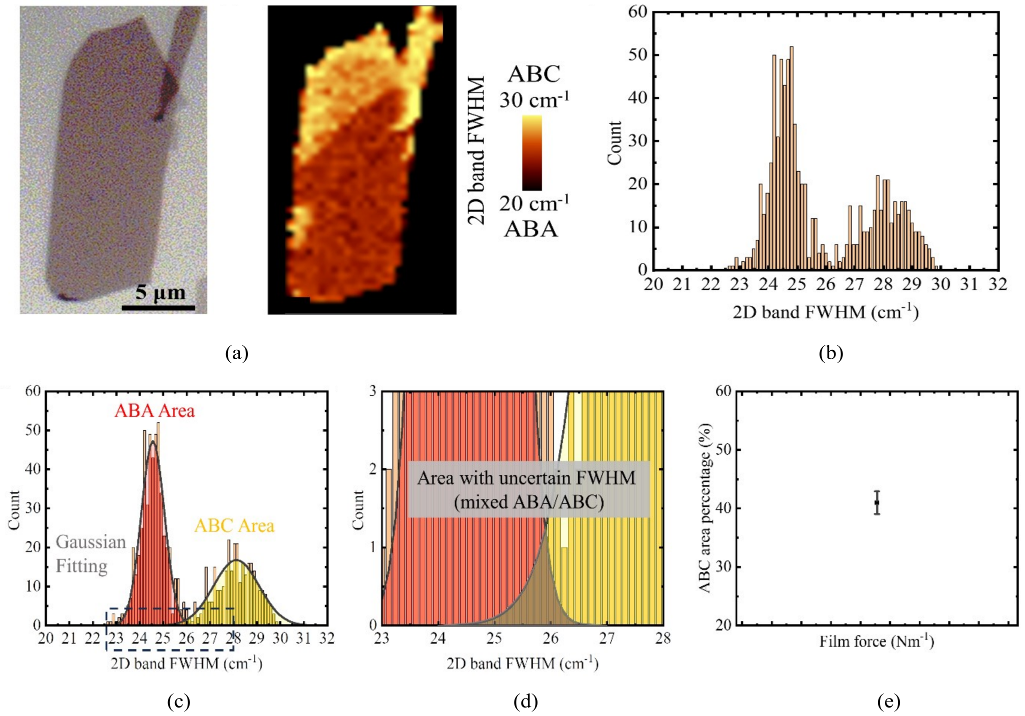

FIGURE S9: (a) Optical image and Raman map of 2D band FWHM of trilayer graphene sample. (b) Histogram plot of the 2D band FWHM distribution extracted from the Raman map. (c) Distribution of 2D band FWHM from the Raman map of trilayer graphene with Gaussian fittings. (d) The overlapped area between the two Gaussians denotes the uncertain FWHM values between ABA and ABC used for error of area calculation. (e) The calculated area percentage and error bars from the Gaussian fits.

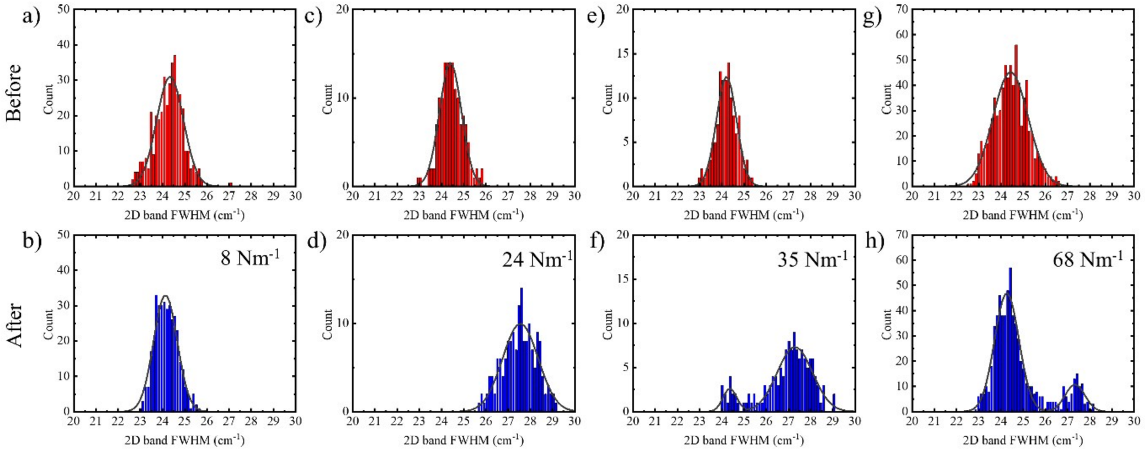

FIGURE S10: (a) and (b) Distribution of 2D band FWHM from the Raman map of unencapsulated trilayer graphene before and after application of 8 Nm-1 stressor. (c) and (d) Distribution of 2D band FWHM from the Raman map of unencapsulated trilayer graphene before and after application of 24 Nm-1 stressor. (e) and (f) Distribution of 2D band FWHM from the Raman map of unencapsulated trilayer graphene before and after application of 35 Nm-1 stressor. (g) and (h) Distribution of 2D band FWHM from the Raman map of unencapsulated trilayer graphene before and after application of 68 N/m stressor.

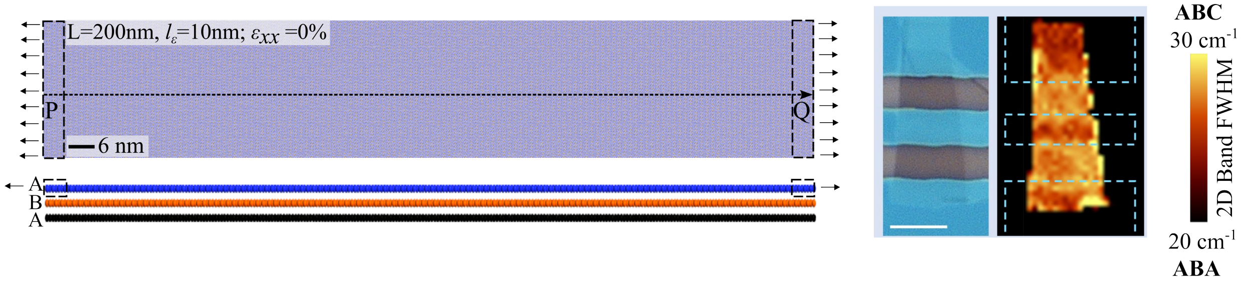

(a)

(b)

FIGURE S11: (a) Atomistic model for molecular statics (MS) simulations to investigate the straining on both sides of the ABA flake (L=200nm). The model incorporates uniaxial tensile strain applied at both edges along the armchair axis, as indicated by black arrows. The tab region, with a width of $l_\epsilon = 10$ nm (5% of the flake length), is shown by the black dotted box. (b) Experimental sample presenting the striped stressor (the blue regions) along with a 2D band optical image of the TLG sample. The grey regions depict the portion of the sample experiencing tension from both edges, subsequently undergoing a stacking transition.

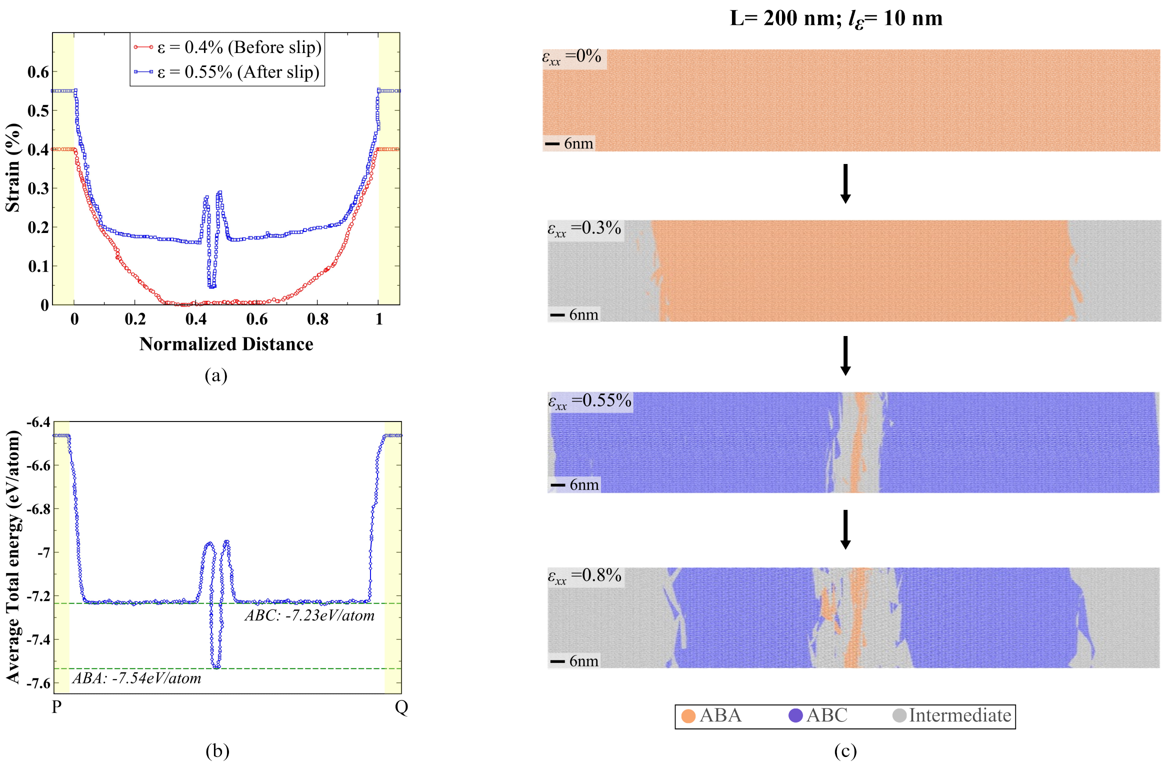

FIGURE S12: (a) Strain profile of the top layer upon straining both edges of the flake. Pre-slippage in-plane strain follows monotonous decay and at slippage ($\epsilon_{xx} = 0.55\%$), strain magnitude plateaus and forms a typical profile. (b) Total energy distribution (TE/atom) profile along path PQ at slippage. The green dotted lines show the TE/atom magnitudes of pristine ABA or ABC systems. (c) Stacking order change with increasing strain magnitudes showing the evolution of ABC domains at slippage strain.

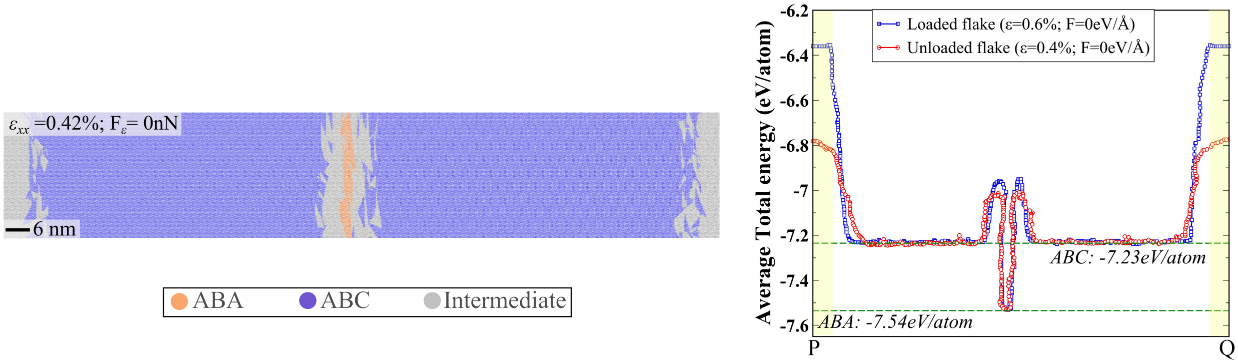

(a) (b)

FIGURE S13: (a) Atomic snapshot with identified sub-domains while the flake is completely unloaded (starting from $\epsilon_{xx} = 0.6\%$). The residual strain magnitude present in the tab region is mentioned, which ultimately helps in preserving the ABC domains. (b) Total

REFERENCES

1. Gui, G., Li, J. & Zhong, J. Band structure engineering of graphene by strain: First-principles calculations. *Physical Review B* **78,** 075435 (2008).

2. Pozzo, M., Alfe, D., Lacovig, P., Hofmann, P., Lizzit, S. & Baraldi, A. Thermal expansion of supported and freestanding graphene: lattice constant versus interatomic distance. *Physical review letters* **106,** 135501 (2011).

3. Wang, K., Qu, C., Wang, J., Ouyang, W., Ma, M. & Zheng, Q. Strain engineering modulates graphene interlayer friction by moiré pattern evolution. *ACS applied materials & interfaces* **11,** 36169–36176 (2019).

4. Ohta, T., Bostwick, A., Seyller, T., Horn, K. & Rotenberg, E. Controlling the electronic structure of bilayer graphene. *Science* **313,** 951–954 (2006).

5. Baroni, S., De Gironcoli, S., Dal Corso, A. & Giannozzi, P. Phonons and related crystal properties from density-functional perturbation theory. *Reviews of modern Physics* **73,** 515 (2001).

6. Putrino, A., Sebastiani, D. & Parrinello, M. Generalized variational density functional perturbation theory. *The Journal of Chemical Physics* **113,** 7102–7109 (2000).

7. Azizimanesh, A., Dey, A., Chowdhury, S. A., Wenner, E., Hou, W., Peña, T., Askari, H. & Wu, S. M. Strain engineering in 2D hBN and graphene with evaporated thin film stressors. *Applied Physics Letters* **123,** 043504. ISSN: 0003-6951 (July 2023).

8. Abraham, J. A., Dey, A. & Kumari, M. Ruthenium-based half Heusler alloys RuTiX (X= Si, Ge, Sn): An FP-LAPW-based analytical study of structural, electronic, elastic, mechanical and transport properties. *International Journal of Modern Physics B* **35,** 2150046 (2021).

9. Abraham, J. A., Sharma, R., Ahmad, S. & Dey, A. DFT investigation on the electronic, optical and thermoelectric properties of novel half-Heusler compounds ScAuX (X= Si, Ge, Sn, Pb) for energy harvesting technologies. *The European Physical Journal Plus* **136,** 1091 (2021).

10. Mahato, M. C., Krishnamurthy, H. & Ramakrishnan, T. Phonon dispersion of crystalline solids from the density-functional theory of freezing. *Physical Review B* **44,** 9944 (1991).

11. Van Troeye, B., Torrent, M. & Gonze, X. Interatomic force constants including the DFT-D dispersion contribution. *Physical Review B* **93,** 144304 (2016).

12. Popov, V. N. Two-phonon Raman bands of bilayer graphene: Revisited. *Carbon* **91,** 436–444 (2015).

13. Frank, O., Mohr, M., Maultzsch, J., Thomsen, C., Riaz, I., Jalil, R., Novoselov, K. S., Tsoukleri, G., Parthenios, J., Papagelis, K., *et al.* Raman 2D-band splitting in graphene: theory and experiment. *ACS nano* **5,** 2231–2239 (2011).

14. Mohr, M., Maultzsch, J. & Thomsen, C. Splitting of the Raman 2 D band of graphene subjected to strain. *Physical Review B* **82,** 201409 (2010).

15. Nika, D. L. & Balandin, A. A. Phonons and thermal transport in graphene and graphene-based materials. *Reports on Progress in Physics* **80,** 036502 (2017).

16. Nika, D. L. & Balandin, A. A. Two-dimensional phonon transport in graphene. *Journal of Physics: Condensed Matter* **24,** 233203 (2012).

17. Warner, J. H., Schäffel, F., Rümmeli, M. H. & Büchner, B. Examining the edges of multi-layer graphene sheets. *Chemistry of Materials* **21,** 2418–2421 (2009).

18. Gupta, A. K., Russin, T. J., Gutiérrez, H. R. & Eklund, P. C. Probing graphene edges via Raman scattering. *ACS nano* **3,** 45–52 (2009).

19. Fujihara, M., Inoue, R., Kurita, R., Taniuchi, T., Motoyui, Y., Shin, S., Komori, F., Maniwa, Y., Shinohara, H. & Miyata, Y. Selective formation of zigzag edges in graphene cracks. *ACS nano* **9,** 9027–9033 (2015).

20. Canny, J. A computational approach to edge detection. *IEEE Transactions on pattern analysis and machine intelligence,* 679–698 (1986).

21. Guo, Y., Liu, C., Yin, Q., Wei, C., Lin, S., Hoffman, T. B., Zhao, Y., Edgar, J., Chen, Q., Lau, S. P., *et al.* Distinctive in-plane cleavage behaviors of two-dimensional layered materials. *ACS nano* **10,** 8980–8988 (2016).

22. Wang, Y.-P., Li, X.-G., Fry, J. N. & Cheng, H.-P. First-principles studies of electric field effects on the electronic structure of trilayer graphene. *Physical Review B* **94,** 165428 (2016).

23. Menezes, M. G., Capaz, R. B. & Louie, S. G. Ab initio quasiparticle band structure of ABA and ABC-stacked graphene trilayers. *Physical Review B* **89,** 035431 (2014).

24. Zollner, K., Gmitra, M. & Fabian, J. Proximity spin-orbit and exchange coupling in ABA and ABC trilayer graphene van der Waals heterostructures. *Physical Review B* **105,** 115126 (2022).

25. Hass, J., Feng, R., Millán-Otoya, J., Li, X., Sprinkle, M., First, P. N., De Heer, W., Conrad, E. & Berger, C. Structural properties of the multilayer graphene/4 H- Si C (000 1
) system as determined by surface x-ray diffraction. *Physical Review B* **75,** 214109 (2007).
